# Supplementary material for: The Impact of Dental Care Programs on Individuals and Their Families: A Scoping Review
Source: Dent J (Basel). 2023 Jan 30;11(2):33. doi: 10.3390/dj11020033 (PMC9954911; doi:10.3390/dj11020033)
Supplement: Supplementary file 1 [file dentistry-11-00033-s001.zip › Supplementary file 1 Search Strategy.pdf]

Supplementary file 1. The search strategy

The following databases were searched: MEDLINE, Embase, and CINAHL. MEDLINE and Embase were searched using the Ovid interface. CINAHL was searched using the EBSCO interface. The MEDLINE search strategy was developed, and peer reviewed using the PRESS standard. The MEDLINE search was then adapted for the other databases.

Searches in MEDLINE, Embase and CINAHL were limited by study design in adherence to the scope of this review. Please see below for the detail inclusion and exclusion criteria for each database. When no appropriate filters needed are available through the databases searched, external pre-tested and published filters by using the suggested filters from InterTASC Information Specialists' Sub-Group Search Filter Resource[1]. In Ovid, Qualitative studies, Case-control studies and Cohort studies filters were adapted from University of Texas Health Science Center at Houston[2]. In CINAHL, search filters were adapted from Wilcaynski's article[3]. Animal studies were excluded by using the animal filters available and key word search in each database. Keywords search were also used to exclude equipment or supplies or dental students or dental schools which are not within the scope of this review.

1. **Ovid MEDLINE: Epub Ahead of Print, In-Process & Other Non-Indexed Citations, Ovid MEDLINE® Daily and Ovid MEDLINE® 1946-Present**

   Language: English

   Subjects: Human

---

[1] ISSG Search Filter Resource [Internet]. Glanville J, Lefebvre C, Wright K, editors. York (UK): The InterTASC Information Specialists' Sub-Group; 2008 [updated 2019 August 12; cited INSERT DATE]. Available from: https://sites.google.com/a/york.ac.uk/issg-search-filters-resource/home

[2] Search Filters for Various Databases [Internet]. The University of Texas Health Science Center at Houston (UTHealth), 2019. [cited 2019 12 13]. Available from: https://libguides.sph.uth.tmc.edu/search_filters

[3] Wilczynski NL, Marks S, Haynes RB. Search strategies for identifying qualitative studies in CINAHL. Qual Health Res., 2007; 17(5):705-10. [cited 2019 12 12]. Available from https://journals.sagepub.com/doi/abs/10.1177/1049732306294515.

Age group: No limitation

Publication year: after 1999

Publication Types: Observational Studies, Systematic Reviews, Meta-analysis

External Filters used: qualitative studies, cohort studies, case control studies.

Exclusion: equipment and supplies, all animal studies

Searched on November 18, 2021

Records retrieved: 34113

Ovid MEDLINE: Epub Ahead of Print, In-Process & Other Non-Indexed Citations, Ovid MEDLINE® Daily and Ovid MEDLINE® <1946-Present>

Search history sorted by search number ascending.

#	Searches
1	exp Dental Care/
2	Comprehensive Dental Care/
3	exp Dental clinics/
4	public health dentistry/ or community dentistry/ or fluoridation/ or health education, dental/
5	exp Mouth rehabilitation/
6	exp Oral diagnosis/
7	exp General practice, dental/
8	exp Education, Dental/
9	exp Practice management, Dental/
10	exp Dental prophylaxis/
11	exp Dental health services/
12	exp Dentistry, Operative/
13	exp Endodontics/
14	exp Esthetics, Dental/

15	exp Infection control, Dental/
16	exp Oral surgical procedures/
17	exp Orthodontics/
18	exp Oral pathology/
19	exp Periodontics/
20	exp Preventive dentistry/
21	exp Prosthodontics/
22	exp Oral surgery/
23	exp Oral medicine/
24	(care adj3 dental).tw,kf.
25	(dental adj3 service*).tw,kf.
26	(dent* adj2 (check-up or health or intervention* or clinic* or treatment* or therap* or program* or practice* or education or procedure* or restorat* regenerative*)).tw,kf.
27	dental stress analys?s.tw,kf.
28	denture*.tw,kf.
29	(oral adj2 care).tw,kf.
30	(dental health adj2 survey*).tw,kf.
31	((decayed or missing or filled) adj3 teeth).tw,kf.
32	(bleeding on probing adj3 gingival).tw,kf.
33	((periodont* or orthodontic* or prosthodont* or endodontic) adj2 (treatment* or procedure* or surger* or care)).tw,kf.
34	((dental or t??th or periodontal) adj1 cleaning).tw,kf.
35	(endodontics or orthodontics or periodontics or prosthodontics).tw,kf.
36	(orthodontia or orthodontolog* or orthodonty).tw,kf.
37	(Orthodontic adj2 (space closure or anchorage* or extrusion*)).tw,kf.
38	(root canal adj1 (therap* or procedure* or obturation* or preparation* or surger*)).tw,kf.
39	((pulpectom* or pulpotom* or apexification* or apex) adj1 excision*).tw,kf.
40	((oral or mouth) adj2 surg*).tw,kf.
41	parotidectom*.tw,kf.
42	((pre prosthetic or preprosthetic) adj1 surger*).tw,kf.

43	(cavity lining* adj1 dental).tw,kf.
44	(varnish* adj2 cavity).tw,kf.
45	(community periodontal index of treatment needs or cpitn).tw,kf.
46	((mouth or oral) adj2 rehabilitation*).tw,kf.
47	((diagnos* or examination*) adj1 (oral or dental)).tw,kf.
48	dental caries activity test*.tw,kf.
49	(dental adj1 pulp test*).tw,kf.
50	((dental or intraoral or orthodontic) adj1 photograph*).tw,kf.
51	((dental or bitewing or tooth) adj1 (radiography or radiovisiography)).tw,kf.
52	(Dental adj1 (x ray or xray)).tw,kf.
53	(fluorescence adj1 quantitative light-induced).tw,kf.
54	(community adj1 dentistry).tw,kf.
55	((dental or periodontal or t??th) adj1 prophylaxis).tw,kf.
56	((supragingival or subgingival or dental or root) adj1 scaling*).tw,kf.
57	((maxillo-mandibular or maxillomandibular or oral or maxillofacial or orthognathic or jaw) adj1 surg*).tw,kf.
58	((maxillo-mandibular or maxillomandibular or oral or maxillofacial or orthognathic) adj2 procedure*).tw,kf.
59	(oral adj1 hygiene).tw,kf.
60	((evidence-based or preventative or cosmetic or operative or prosthetic or reparative) adj2 dentistry).tw,kf.
61	(dental adj1 (esthetic* or aesthetic* or floss or device* or scaling* or brace* or hygiene* or polishing or radiology or bonding or soldering* or prosthetic*)).tw,kf.
62	((dental or tooth) adj3 filling*).tw,kf.
63	((dental or tooth or caries) adj2 prevention).tw,kf.
64	((tooth or dental) adj1 (an?esthesia or an?esthetic* or casting*)).tw,kf.
65	Jaw relation record*.tw,kf.
66	(Sinus adj1 (floor augmentation* or lifting*)).tw,kf.
67	(dental adj2 (implant* or prosthes?s)).tw,kf.
68	((dental or enamel) adj1 microabrasion*).tw,kf.
69	(t??th adj1 (bleaching or whitening or replantation* or reimplantation* or extrusion* or uprighting* or remineralization* or polishing or brushing or restoration* or surger* or inlay* or preparation* or removal* or resection*)).tw,kf.

70	(Dental adj1 (reimplantation* or replantation* or reinclusion*)).tw,kf.
71	(dental adj1 infection adj1 control*).tw,kf.
72	(apicoectom* or gingivectom* or gingivoplast* or glossectom*).tw,kf.
73	((tongue or lingual) adj1 (extirpation* or resection*)).tw,kf.
74	((jaw or maxillomandibular) adj1 fixation*).tw,kf.
75	(mandibula* adj1 (advancement* or reconstruction* or restoration* or resection*)).tw,kf.
76	(mandible ostectom* or mandibulectom*).tw,kf.
77	((maxillofacial or mandibular) adj1 (prosthes?s or impant*)).tw,kf.
78	((upper jaw or maxilla*) adj1 resection).tw,kf.
79	maxillectom*.tw,kf.
80	genioplast*.tw,kf.
81	(chin adj2 (correction* or reconstruction * or reduction* or surger* or operation*)).tw,kf.
82	(chinplast* or mentoplast*).tw,kf.
83	((maxillary or mandibular or alveolar) adj1 ridge augmentation*).tw,kf.
84	(alveolectom* or alveoloplast* or vestibuloplast*).tw,kf.
85	((endosseous or subperiosteal) adj2 implant*).tw,kf.
86	(alveolar adj2 graft*).tw,kf.
87	((le fort or lefort or maxilla* or jaw or mandib*) adj2 osteotom*).tw,kf.
88	(Le fort adj1 operation*).tw,kf.
89	(sagittal split adj2 osteotom*).tw,kf.
90	(sinus adj2 augmentation*).tw,kf.
91	((t??th or serial) adj1 extraction*).tw,kf.
92	Exodont*.tw,kf.
93	(Molar adj1 (amputation* or extraction*)).tw,kf.
94	odontectom*.tw,kf.
95	(uvulopharyngopalatoplast* or uvulopalatoplast* or palatouvulopharyngoplast* or pharyngouvulopalatoplast*).tw,kf.
96	(dental adj1 (internal or marginal) adj1 adaptation*).tw,kf.
97	(dental adj1 internal adj1 fit*).tw,kf.
98	(mandibular adj1 advancement).tw,kf.

99	(occlusal adj1 (splint* or adjustment* or equilibration*)).tw,kf.
100	((fixed or activator or extraoral) adj2 appliance*).tw,kf.
101	((fixed or bonded or permanent) adj1 retainer*).tw,kf.
102	((clear aligner or herbst or bimler or andresen) adj1 appliance*).tw,kf.
103	(frankel adj2 regulator*).tw,kf.
104	kinetor*.tw,kf.
105	((function or harvold) adj1 activator*).tw,kf.
106	(jasper adj1 jumper*).tw,kf.
107	bionator*.tw,kf.
108	invisalign*.tw,kf.
109	(crown adj1 lengthening).tw,kf.
110	(forced adj2 eruption*).tw,kf.
111	((maxillary or palatal) adj1 expansion*).tw,kf.
112	palatoplast*.tw,kf.
113	(palate adj1 (plast* or reconstruction* or operation*)).tw,kf.
114	(tooth adj2 (movement* or intrusion* or depression* or care)).tw,kf.
115	((oral or maxillofacial) adj3 patholog*).tw,kf.
116	(periodontal adj1 (medicine*or prosthes?s or splint* or dressing* or prevention*)).tw,kf.
117	(periodontal adj2 (tissue regeneration* or debridement*)).tw,kf.
118	((dental or mouth or t??th) adj1 debridement*).tw,kf.
119	(root adj1 planing*).tw,kf.
120	((subgingival or gingival) adj1 (curettage* or retraction*)).tw,kf.
121	fluoridation*.tw,kf.
122	(mouth adj1 (protect* or guard)).tw,kf.
123	stomatolog*.tw,kf.
124	(medicine adj1 oral).tw,kf.
125	or/1-124
126	exp Health promotion/ or Public health/ or exp Patient education as topic/
127	((promotion* or campaign* or program*) adj2 health).tw,kf.
128	(program* adj2 wellness).tw,kf.

129	(head start adj1 program*).tw,kf.
130	(Pathology or pathological or telepathology).tw,kf.
131	((public or deliver* or system*) adj1 (health care or healthcare)).tw,kf.
132	or/126-131
133	limit 132 to dentistry journals
134	exp Dentistry/
135	132 and 134
136	133 or 135
137	125 or 136
138	exp Health status/
139	exp Oral health/
140	exp Critical illness/
141	exp Disease/
142	exp Comorbidity/
143	exp Cohort effect/
144	Pain/
145	exp Acute Pain/
146	exp Chronic Pain/
147	Facial Pain/
148	exp Pain, Postoperative/
149	exp Pain, Procedural/
150	exp Pain, Referred/
151	exp Mental health/
152	Mental Disorders/
153	exp Anxiety Disorders/
154	"Feeding and Eating Disorders"/
155	exp Mood Disorders/
156	Sleep/
157	exp Apnea/
158	exp Feasibility studies/

159	Adaptation, Psychological/
160	exp Emotional Adjustment/
161	exp Feedback, Psychological/
162	exp Orientation/
163	Behavior/
164	Social behavior/
165	exp Social adjustment/
166	exp Social skills/
167	exp Shyness/
168	exp Rejection/
169	exp Personal satisfaction/
170	Communication/
171	exp Verbal behavior/
172	Nonverbal Communication/
173	Facial Expression/
174	exp Smiling/
175	Behavioral Symptoms/
176	exp Psychological stress/
177	exp Habits/
178	Emotions/
179	Anxiety/
180	exp Dental Anxiety/
181	Motivation/
182	exp Food Deprivation/
183	Personality/
184	Personality Development/
185	exp Self Concept/
186	exp Quality of life/
187	exp Sensation disorders/
188	exp Eating/

189	exp Body Mass Index/
190	Body Size/
191	exp Body Weight/
192	exp Chronic disease/
193	exp Saliva/
194	exp Sepsis/
195	exp Value of life/
196	exp Family health/
197	exp family relations/
198	exp Interpersonal relations/
199	exp Social isolation/
200	exp periodontal diseases/
201	tooth diseases/ or exp dental leakage/ or dentin sensitivity/ or fluorosis, dental/ or focal infection, dental/ or exp mouth, edentulous/ or exp tooth demineralization/ or tooth discoloration/ or tooth loss/ or toothache/
202	or/138-201
203	((health or disease*) adj2 (oral or mouth or dental)).tw,kf.
204	((participat* or involvement* or action*) adj1 (community or consumer or public)).tw,kf.
205	(patient adj2 outcome measures).tw,kf.
206	(self report or patient acuity or illness index or performance status or sickness impact profile or geriatric assessment).tw,kf.
207	((dysfunction or physiology) adj score*).tw,kf.
208	((mass or multiphasic or neonatal) adj screening*).tw,kf.
209	((public health or population or sentinel or behavioral risk factor) adj surveillance).tw,kf.
210	((oral or health) adj3 complication*).tw,kf.
211	((health or functional) adj2 (level* or status*)).tw,kf.
212	(health disparity or physical mobility).tw,kf.
213	oral health.tw,kf.
214	(oral adj6 life).tw,kf.
215	(health adj2 social determinant*).tw,kf.

216	(hrqol or (quality adj3 life)).tw,kf.
217	(appraisal* adj1 health risk).tw,kf.
218	(health status adj1 (index* or indicator* or indices)).tw,kf.
219	(apache or (acute physiology and chronic health evaluation)).tw,kf.
220	(acuit* adj1 patient).tw,kf.
221	(impact profile* adj1 sickness).tw,kf.
222	(cost* adj2 (disease* or illness or sickness)).tw,kf.
223	(critical adj1 ill*).tw,kf.
224	comorbidity.tw,kf.
225	multimorbidity.tw,kf.
226	((cohort or generation) adj1 effect*).tw,kf.
227	ache*.tw,kf.
228	(physical adj1 suffering*).tw,kf.
229	physical functioning.tw,kf.
230	vitality.tw,kf.
231	((physical or psychic) adj health).tw,kf.
232	(postherpetic adj1 neuralgia).tw,kf.
233	(pain* adj2 (nociceptive or tissue or somatic or visceral or postoperative or post operation or procedural or referred)).tw,kf.
234	((sensation* or limb* or pain) adj2 phantom).tw,kf.
235	((acute or chronic or face or facial or craniofacial or myofacial or orofacial or physical or splitting or radiating or migratory or crushing or burning or malignant) adj2 pain*).tw,kf.
236	(oral adj3 pain).tw,kf.
237	chronic pain.tw,kf.
238	(pain adj2 (management or measurement*)).tw,kf.
239	(adl or self-care or worry or depression*).tw,kf.
240	(activit* adj3 (daily living or limitation*)).tw,kf.
241	((care or administration* medication* or management or neglect or help or treatment*) adj1 self).tw,kf.
242	(selfcare or selfmanagement).tw,kf.
243	aging in place.tw,kf.

244	(community adj1 dwelling*).tw,kf.
245	(independent adj1 living).tw,kf.
246	(participation adj1 social).tw,kf.
247	(depressive adj1 symptom*).tw,kf.
248	(diagnosis adj1 psychiatric).tw,kf.
249	((effort or distress) adj1 syndrome).tw,kf.
250	((disorder* or obsessive) adj1 hoarding).tw,kf.
251	((disorder* or neuros?s) adj1 phobic).tw,kf.
252	(mood adj (disturbance* or change)).tw,kf.
253	(fear or frustration or happ* or hope* or helplessness or anger or pleasure or unhapp* or motivation or aspiration* or goal*).tw,kf.
254	(affective adj (illness or neuros?s or psychos?s)).tw,kf.
255	(cyclothymic adj1 personalit*).tw,kf.
256	(depression* adj1 (endogenous or neurotic or unipolar or postnatal or post-partum)).tw,kf.
257	(depressive adj1 (syndrome* or disorders)).tw,kf.
258	((disorder or depression) adj3 dysthymic).tw,kf.
259	((dental or t??th) adj1 (grinding or clenching)).tw,kf.
260	(insufficient adj1 sleep syndrome*).tw,kf.
261	((health or condition or disorder*) adj2 (mental or behavio?r*)).tw,kf.
262	(mental adj (hygiene or care or factor or help or service or state or status)).tw,kf.
263	(mental adj1 (disease or illness)).tw,kf.
264	(mental adj (abnormality or change or confusion or defect or disturbance or insufficiency or symptom)).tw,kf.
265	((patient or personal) adj2 satisfaction).tw,kf.
266	(patient* adj2 preference*).tw,kf.
267	(anxiety* or hypervigilance or nervousness or odontophobia* or uncomfortable or catastrophizing or psychasthenia).tw,kf.
268	(apnea* or apnoea or parasomnia*).tw,kf.
269	(hypopnea* adj2 sleep).tw,kf.
270	(feasibility adj1 stud*).tw,kf.

271	smiling*.tw,kf.
272	((perception* or esteem* or confidence or concept or awareness or confrontation or image or rating or representation or actualization or disclosure or transcendence) adj1 self).tw,kf.
273	(Selfconcept or ego or Personal appearance or professional image or Superego or sense of coherence).tw,kf.
274	((impairment* or disorder* or disability* or disturbance) adj1 (vision or visual)).tw,kf.
275	(Visual adj (illusion or hallucination)).tw,kf.
276	((sensation or sensory or senses) adj1 (disorder* or impairment*)).tw,kf.
277	(abnormal sensation or dizziness).tw,kf.
278	(hearing adj2 (impairment* or loss or disorder*)).tw,kf.
279	hypoacus?s.tw,kf.
280	((bilateral or acquired or prelingual) adj1 deaf*).tw,kf.
281	((extreme or complete) adj1 hearing loss).tw,kf.
282	deaf mutism.tw,kf.
283	((behavior?r or interaction or expression or interaction or communication) adj1 verbal).tw,kf.
284	(abilit* adj2 (speak or eat or sleep)).tw,kf.
285	(((eating or ingestion) adj2 disorder*) or ednos).tw,kf.
286	(food adj1 intake).tw,kf.
287	(drinking* or water intake).tw,kf.
288	(chewing or mastication or obesity or habit* or overweight).tw,kf.
289	(finger sucking or fingersucking).tw,kf.
290	(nail biting or nailbiting).tw,kf.
291	(Onychophagy or tongue habits).tw,kf.
292	(body adj3 measure*).tw,kf.
293	(((body mass or quetelet*) adj1 index) or BMI).tw,kf.
294	(body adj1 (weight* or image)).tw,kf.
295	(leanness or thinness or underweight).tw,kf.
296	(chronic adj2 (disease* or ill* or condition*)).tw,kf.
297	((medical or oral) adj4 condition*).tw,kf.
298	((dental plaque or dmf or gingival or periodontal) adj1 (index* or indices)).tw,kf.

299	(oral hygiene adj1 (index* or indices)).tw,kf.
300	(dentin* adj1 secondary).tw,kf.
301	(pulpitis or pulpitides).tw,kf.
302	(inflammation* adj1 endodontic).tw,kf.
303	(t??th adj1 (endodontically treated or nonvital or devitalized or pulpless)).tw,kf.
304	((sensitivit* or hypersensitivit*) adj1 (dentin or dentine or t??th)).tw,kf.
305	(((focal adj1 infection*) or fluoros?s or overjet) adj1 dental).tw,kf.
306	(mottled adj1 enamel*).tw,kf.
307	((t??th or dentoalveolar or dental) adj2 ankylos?s).tw,kf.
308	((decay or caries or fissure*) adj2 dental).tw,kf.
309	(caries adj2 stage*).tw,kf.
310	(white adj1 spot*).tw,kf.
311	(dental adj3 spot*).tw,kf.
312	(carious adj2 dentin*).tw,kf.
313	((cary or caries) adj2 (cervical or root)).tw,kf.
314	(odontalgia* or toothache* or tooth pain).tw,kf.
315	(disease* adj2 non communicable).tw,kf.
316	non infectious disease*.tw,kf.
317	(dental adj1 plaque).tw,kf.
318	sepsis.tw,kf.
319	((periodontal or periapical) adj1 disease*).tw,kf.
320	(disease* adj1 gingival).tw,kf.
321	(periodontiti* or periodontos?s or pericementitis or gingiviti* or fusospirillos?s or pericoroniti*).tw,kf.
322	(pocket* adj1 gingival).tw,kf.
323	(gingiv* adj2 (atroph* or recession*)).tw,kf.
324	((alveolar or periodont*) adj2 (bone loss* or atroph* or resorption)).tw,kf.
325	(periodontal attachment adj1 loss).tw,kf.
326	((periodontal or gingival) adj1 (abscess* or pocket*)).tw,kf.
327	suffering*.tw,kf.

328	((psychologic* or life or emotional or disorder* or management or mental or psychic or psycho social) adj1 stress*).tw,kf.
329	(emotional adj (factor or response or status or structure)).tw,kf.
330	emotion*.tw,kf.
331	((Mental or psychic) adj1 Tension).tw,kf.
332	schizotypal personality.tw,kf.
333	(personality adj1 (characteristic or pattern or structure or type)).tw,kf.
334	(burn out or burnout).tw,kf.
335	((work place or workplace or work or job or occupational or professional) adj2 stress*).tw,kf.
336	(saliva* or spittle).tw,kf.
337	(blood adj1 poisoning*).tw,kf.
338	(seps?s or septicemia* or bacteremia* or fungemia*).tw,kf.
339	((endotoxic or septic or toxic) adj1 shock).tw,kf.
340	((right or respect or sanctit* or value) adj2 life).tw,kf.
341	(family adj1 planning).tw,kf.
342	(family adj1 allowance*).tw,kf.
343	(family adj2 health).tw,kf.
344	(family adj5 life).tw,kf.
345	(family adj3 well*).tw,kf.
346	(family adj1 (dynamic* or relation* or interaction* or outcome* or histor*)).tw,kf.
347	((family or interparental or marital) adj1 conflict*).tw,kf.
348	(family adj2 relation*).tw,kf.
349	or/203-348
350	202 or 349
351	137 and 350
352	limit 351 to animals
353	351 not 352
354	limit 353 to (english language and yr="1999 -Current" and journal article)
355	limit 354 to (meta analysis or observational study or "systematic review")

356	((("semi-structured" or semistructured or unstructured or informal or "in-depth" or indepth or "face-to-face" or structured or guide) adj3 (interview* or discussion* or questionnaire*)) or (focus group* or qualitative or ethnograph* or fieldwork or "field work" or "key informant")).ti,ab. or interviews as topic/ or focus groups/ or narration/ or qualitative research/
357	Case-Control Studies/ or Control Groups/ or Matched-Pair Analysis/ or ((case* adj5 control*) or (case adj3 comparison*) or control group*).ti,ab.
358	cohort studies/ or longitudinal studies/ or follow-up studies/ or prospective studies/ or retrospective studies/ or cohort.ti,ab. or longitudinal.ti,ab. or prospective.ti,ab. or retrospective.ti,ab.
359	356 or 357 or 358
360	354 and 359
361	355 or 360
362	(rats or rat or mice or dog* or pig* or horse* or dog* or mouse or rabbit* or Animal*).tw,kf.
363	(Equipment* or instrument* or device* or supply or supplies or t??th brush* or t??thbrush or tooth paste or toothpaste or versus).ti.
364	(dental adj1 (student* or school* or university or universities or college*)).tw,kf.
365	362 or 363 or 364
366	361 not 365

Key

/ = indexing term (MeSH heading)

exp = exploded indexing term (MeSH heading)

$ = truncation

tw = text word search in title or abstract fields

kf = terms in author provided keyword

pt = publication type

adjn = terms within (n-1) words of each other (any order)

2. **EMBASE:**

Language: English

Subjects: Not animals

Age group: No limitation

Publication year: after 1999

Clinical Queries: "qualitative (best balance of sensitivity and specificity)"

External Filters used: cohort studies, case control studies

EBM-Evidence Based Medicine: all

Exclusion: equipment and supplies, all animal studies

Searched on November 18, 2021

Records retrieved: 51195

Embase Classic + Embase <1947 to 2021 November 18>

Search history sorted by search number ascending.

#	Searches
1	exp dental procedure/
2	dental clinics/
3	dental health education/
4	exp Dental prophylaxis/
5	operative dentistry/
6	Endodontics/
7	Orthodontics/
8	Periodontics/
9	Preventive dentistry/

10	Prosthodontics/
11	exp Oral surgery/
12	(care adj3 dental).tw,kw.
13	(dental adj3 service*).tw,kw.
14	(dent* adj2 (check-up or health or intervention* or clinic* or treatment* or therap* or program* or practice* or education or procedure* or restorat* regenerative*)).tw,kw.
15	dental stress analys?s.tw,kw.
16	denture*.tw,kw.
17	(oral adj2 care).tw,kw.
18	(dental health adj2 survey*).tw,kw.
19	((decayed or missing or filled) adj3 teeth).tw,kw.
20	(bleeding on probing adj3 gingival).tw,kw.
21	((periodont* or orthodontic* or prosthodont* or endodontic) adj2 (treatment* or procedure* or surger* or care)).tw,kw.
22	((dental or t??th or periodontal) adj1 cleaning).tw,kw.
23	(endodontics or orthodontics or periodontics or prosthodontics).tw,kw.
24	(orthodontia or orthodontolog* or orthodonty).tw,kw.
25	(Orthodontic adj2 (space closure or anchorage* or extrusion*)).tw,kw.
26	(root canal adj1 (therap* or procedure* or obturation* or preparation* or surger*)).tw,kw.
27	((pulpectom* or pulpotom* or apexification* or apex) adj1 excision*).tw,kw.
28	((oral or mouth) adj2 surg*).tw,kw.
29	parotidectom*.tw,kw.
30	((pre prosthetic or preprosthetic) adj1 surger*).tw,kw.
31	(cavity lining* adj1 dental).tw,kw.
32	(varnish* adj2 cavity).tw,kw.
33	(community periodontal index of treatment needs or cpitn).tw,kw.
34	((mouth or oral) adj2 rehabilitation*).tw,kw.
35	((diagnos* or examination*) adj1 (oral or dental)).tw,kw.
36	dental caries activity test*.tw,kw.
37	(dental adj1 pulp test*).tw,kw.

38	((dental or intraoral or orthodontic) adj1 photograph*).tw,kw.
39	((dental or bitewing or tooth) adj1 (radiography or radiovisiography)).tw,kw.
40	(Dental adj1 (x ray or xray)).tw,kw.
41	(fluorescence adj1 quantitative light-induced).tw,kw.
42	(community adj1 dentistry).tw,kw.
43	((dental or periodontal or t??th) adj1 prophylaxis).tw,kw.
44	((supragingival or subgingival or dental or root) adj1 scaling*).tw,kw.
45	((maxillo-mandibular or maxillomandibular or oral or maxillofacial or orthognathic or jaw) adj1 surg*).tw,kw.
46	((maxillo-mandibular or maxillomandibular or oral or maxillofacial or orthognathic) adj2 procedure*).tw,kw.
47	(oral adj1 hygiene).tw,kw.
48	((evidence-based or preventative or cosmetic or operative or prosthetic or reparative) adj2 dentistry).tw,kw.
49	(dental adj1 (esthetic* or aesthetic* or floss or device* or scaling* or brace* or hygiene* or polishing or radiology or bonding or soldering* or prosthetic*)).tw,kw.
50	((dental or tooth) adj3 filling*).tw,kw.
51	((dental or tooth or caries) adj2 prevention).tw,kw.
52	((tooth or dental) adj1 (an?esthesia or an?esthetic* or casting*)).tw,kw.
53	Jaw relation record*.tw,kw.
54	(Sinus adj1 (floor augmentation* or lifting*)).tw,kw.
55	(dental adj2 (implant* or prosthes?s)).tw,kw.
56	((dental or enamel) adj1 microabrasion*).tw,kw.
57	(t??th adj1 (bleaching or whitening or replantation* or reimplantation* or extrusion* or uprighting* or remineralization* or polishing or brushing or restoration* or surger* or inlay* or preparation* or removal* or resection*)).tw,kw.
58	(Dental adj1 (reimplantation* or replantation* or reinclusion*)).tw,kw.
59	(dental adj1 infection adj1 control*).tw,kw.
60	(apicoectom* or gingivectom* or gingivoplast* or glossectom*).tw,kw.
61	((tongue or lingual) adj1 (extirpation* or resection*)).tw,kw.
62	((jaw or maxillomandibular) adj1 fixation*).tw,kw.

63	(mandibula* adj1 (advancement* or reconstruction* or restoration* or resection*)).tw,kw.
64	(mandible ostectom* or mandibulectom*).tw,kw.
65	((maxillofacial or mandibular) adj1 (prosthes?s or impant*)).tw,kw.
66	((upper jaw or maxilla*) adj1 resection).tw,kw.
67	maxillectom*.tw,kw.
68	genioplast*.tw,kw.
69	(chin adj2 (correction* or reconstruction * or reduction* or surger* or operation*)).tw,kw.
70	(chinplast* or mentoplast*).tw,kw.
71	((maxillary or mandibular or alveolar) adj1 ridge augmentation*).tw,kw.
72	(alveolectom* or alveoloplast* or vestibuloplast*).tw,kw.
73	((endosseous or subperiosteal) adj2 implant*).tw,kw.
74	(alveolar adj2 graft*).tw,kw.
75	((le fort or lefort or maxilla* or jaw or mandib*) adj2 osteotom*).tw,kw.
76	(Le fort adj1 operation*).tw,kw.
77	(sagittal split adj2 osteotom*).tw,kw.
78	(sinus adj2 augmentation*).tw,kw.
79	((t??th or serial) adj1 extraction*).tw,kw.
80	Exodont*.tw,kw.
81	(Molar adj1 (amputation* or extraction*)).tw,kw.
82	odontectom*.tw,kw.
83	(uvulopharyngopalatoplast* or uvulopalatoplast* or palatouvulopharyngoplast* or pharyngouvulopalatoplast*).tw,kw.
84	(dental adj1 (internal or marginal) adj1 adaptation*).tw,kw.
85	(dental adj1 internal adj1 fit*).tw,kw.
86	(mandibular adj1 advancement).tw,kw.
87	(occlusal adj1 (splint* or adjustment* or equilibration*)).tw,kw.
88	((fixed or activator or extraoral) adj2 appliance*).tw,kw.
89	((fixed or bonded or permanent) adj1 retainer*).tw,kw.
90	((clear aligner or herbst or bimler or andresen) adj1 appliance*).tw,kw.
91	(frankel adj2 regulator*).tw,kw.

92	kinetor*.tw,kw.
93	((function or harvold) adj1 activator*).tw,kw.
94	(jasper adj1 jumper*).tw,kw.
95	bionator*.tw,kw.
96	invisalign*.tw,kw.
97	(crown adj1 lengthening).tw,kw.
98	(forced adj2 eruption*).tw,kw.
99	((maxillary or palatal) adj1 expansion*).tw,kw.
100	palatoplast*.tw,kw.
101	(palate adj1 (plast* or reconstruction* or operation*)).tw,kw.
102	(tooth adj2 (movement* or intrusion* or depression* or care)).tw,kw.
103	((oral or maxillofacial) adj3 patholog*).tw,kw.
104	(periodontal adj1 (medicine*or prosthes?s or splint* or dressing* or prevention*)).tw,kw.
105	(periodontal adj2 (tissue regeneration* or debridement*)).tw,kw.
106	((dental or mouth or t??th) adj1 debridement*).tw,kw.
107	(root adj1 planing*).tw,kw.
108	((subgingival or gingival) adj1 (curettage* or retraction*)).tw,kw.
109	fluoridation*.tw,kw.
110	(mouth adj1 (protect* or guard)).tw,kw.
111	stomatolog*.tw,kw.
112	(medicine adj1 oral).tw,kw.
113	or/1-112
114	exp Health promotion/
115	exp Infection control/
116	exp pathology/
117	Public health/
118	((promotion* or campaign* or program*) adj2 health).tw,kw.
119	(program* adj2 wellness).tw,kw.
120	(head start adj1 program*).tw,kw.
121	(Pathology or pathological or telepathology).tw,kw.

122	((public or deliver* or system*) adj1 (health care or healthcare)).tw,kw.
123	or/114-122
124	exp dentistry/
125	123 and 124
126	113 or 125
127	exp Health status/
128	exp Oral health/
129	exp Critical illness/
130	exp Disease/
131	exp Comorbidity/
132	exp Cohort effect/
133	Pain/
134	exp Chronic Pain/
135	face pain/
136	exp Postoperative pain/
137	exp Procedural pain/
138	exp Referred pain/
139	exp Mental health/
140	mental disease/
141	exp Anxiety Disorders/
142	Eating Disorders/
143	exp Mood Disorders/
144	Sleep Disorders/
145	Sleep Deprivation/
146	exp Bruxism/
147	exp drug dependence/
148	Sleep/
149	apnea/ or apnea attack/ or apparent life threatening event/ or central sleep apnea syndrome/
150	Feasibility studies/
151	exp coping behavior/

152	psychological adjustment/
153	Feedback, Psychological/
154	exp Orientation/
155	Behavior/
156	Shyness/
157	exp satisfaction/
158	interpersonal communication/
159	exp Verbal behavior/
160	nonverbal communication/ or exp facial expression/
161	exp health behavior/
162	Habit/
163	exp Emotion/
164	Motivation/
165	exp Food Deprivation/
166	Personality/
167	exp Self Concept/
168	exp Quality of life/
169	exp Eating/
170	Body Mass/
171	Body Size/
172	exp Body Weight/
173	exp Chronic disease/
174	Saliva/
175	Sepsis/
176	Family health/
177	exp Family relation/
178	exp Social Isolation/
179	mouth diseases/ or mouth infection/ or xerostomia/ or exp lip diseases/ or exp oral fistula/ or mouth ulcer/ or exp tongue disease/

180	tooth disease/ or exp dental caries/ or dental fluorosis/ or dentin sensitivity/ or edentulousness/ or exp periodontal disease/ or tooth discoloration/ or exp tooth infection/ or tooth pain/ or tooth pulp disease/
181	((health or disease*) adj2 (oral or mouth or dental)).tw,kw.
182	((participat* or involvement* or action*) adj1 (community or consumer or public)).tw,kw.
183	(patient adj2 outcome measures).tw,kw.
184	(self report or patient acuity or illness index or performance status or sickness impact profile or geriatric assessment).tw,kw.
185	((dysfunction or physiology) adj score*).tw,kw.
186	((mass or multiphasic or neonatal) adj screening*).tw,kw.
187	((public health or population or sentinel or behavioral risk factor) adj surveillance).tw,kw.
188	((oral or health) adj3 complication*).tw,kw.
189	((health or functional) adj2 (level* or status*)).tw,kw.
190	(health disparity or physical mobility).tw,kw.
191	oral health.tw,kw.
192	(oral adj6 life).tw,kw.
193	(health adj2 social determinant*).tw,kw.
194	(hrqol or (quality adj3 life)).tw,kw.
195	(appraisal* adj1 health risk).tw,kw.
196	(health status adj1 (index* or indicator* or indices)).tw,kw.
197	(apache or (acute physiology and chronic health evaluation)).tw,kw.
198	(acuit* adj1 patient).tw,kw.
199	(impact profile* adj1 sickness).tw,kw.
200	(cost* adj2 (disease* or illness or sickness)).tw,kw.
201	(critical adj1 ill*).tw,kw.
202	comorbidity.tw,kw.
203	multimorbidity.tw,kw.
204	((cohort or generation) adj1 effect*).tw,kw.
205	ache*.tw,kw.
206	(physical adj1 suffering*).tw,kw.

207	physical functioning.tw,kw.
208	vitality.tw,kw.
209	((physical or psychic) adj health).tw,kw.
210	(postherpetic adj1 neuralgia).tw,kw.
211	(pain* adj2 (nociceptive or tissue or somatic or visceral or postoperative or post operation or procedural or referred)).tw,kw.
212	((sensation* or limb* or pain) adj2 phantom).tw,kw.
213	((acute or chronic or face or facial or craniofacial or myofacial or orofacial or physical or splitting or radiating or migratory or crushing or burning or malignant) adj2 pain*).tw,kw.
214	(oral adj3 pain).tw,kw.
215	chronic pain.tw,kw.
216	(pain adj2 (management or measurement*)).tw,kw.
217	(adl or self-care or worry or depression*).tw,kw.
218	(activit* adj3 (daily living or limitation*)).tw,kw.
219	((care or administration* medication* or management or neglect or help or treatment*) adj1 self).tw,kw.
220	(selfcare or selfmanagement).tw,kw.
221	aging in place.tw,kw.
222	(community adj1 dwelling*).tw,kw.
223	(independent adj1 living).tw,kw.
224	(participation adj1 social).tw,kw.
225	(depressive adj1 symptom*).tw,kw.
226	(diagnosis adj1 psychiatric).tw,kw.
227	((effort or distress) adj1 syndrome).tw,kw.
228	((disorder* or obsessive) adj1 hoarding).tw,kw.
229	((disorder* or neuros?s) adj1 phobic).tw,kw.
230	(mood adj (disturbance* or change)).tw,kw.
231	(fear or frustration or happ* or hope* or helplessness or anger or pleasure or unhapp* or motivation or aspiration* or goal*).tw,kw.
232	(affective adj (illness or neuros?s or psychos?s)).tw,kw.

233	(cyclothymic adj1 personalit*).tw,kw.
234	(depression* adj1 (endogenous or neurotic or unipolar or postnatal or post-partum)).tw,kw.
235	(depressive adj1 (syndrome* or disorders)).tw,kw.
236	((disorder or depression) adj3 dysthymic).tw,kw.
237	((dental or t??th) adj1 (grinding or clenching)).tw,kw.
238	(insufficient adj1 sleep syndrome*).tw,kw.
239	((health or condition or disorder*) adj2 (mental or behavio?r*)).tw,kw.
240	(mental adj (hygiene or care or factor or help or service or state or status)).tw,kw.
241	(mental adj1 (disease or illness)).tw,kw.
242	(mental adj (abnormality or change or confusion or defect or disturbance or insufficiency or symptom)).tw,kw.
243	((patient or personal) adj2 satisfaction).tw,kw.
244	(patient* adj2 preference*).tw,kw.
245	(anxiety* or hypervigilance or nervousness or odontophobia* or uncomfortable or catastrophizing or psychasthenia).tw,kw.
246	(apnea* or apnoea or parasomnia*).tw,kw.
247	(hypopnea* adj2 sleep).tw,kw.
248	(feasibility adj1 stud*).tw,kw.
249	smiling*.tw,kw.
250	((perception* or esteem* or confidence or concept or awareness or confrontation or image or rating or representation or actualization or disclosure or transcendence) adj1 self).tw,kw.
251	(Selfconcept or ego or Personal appearance or professional image or Superego or sense of coherence).tw,kw.
252	((impairment* or disorder* or disability* or disturbance) adj1 (vision or visual)).tw,kw.
253	(Visual adj (illusion or hallucination)).tw,kw.
254	((sensation or sensory or senses) adj1 (disorder* or impairment*)).tw,kw.
255	(abnormal sensation or dizziness).tw,kw.
256	(hearing adj2 (impairment* or loss or disorder*)).tw,kw.
257	hypoacus?s.tw,kw.
258	((bilateral or acquired or prelingual) adj1 deaf*).tw,kw.

259	((extreme or complete) adj1 hearing loss).tw,kw.
260	deaf mutism.tw,kw.
261	((behavior?r or interaction or expression or interaction or communication) adj1 verbal).tw,kw.
262	(abilit* adj2 (speak or eat or sleep)).tw,kw.
263	(((eating or ingestion) adj2 disorder*) or ednos).tw,kw.
264	(food adj1 intake).tw,kw.
265	(drinking* or water intake).tw,kw.
266	(chewing or mastication or obesity or habit* or overweight).tw,kw.
267	(finger sucking or fingersucking).tw,kw.
268	(nail biting or nailbiting).tw,kw.
269	(Onychophagy or tongue habits).tw,kw.
270	(body adj3 measure*).tw,kw.
271	(((body mass or quetelet*) adj1 index) or BMI).tw,kw.
272	(body adj1 (weight* or image)).tw,kw.
273	(leanness or thinness or underweight).tw,kw.
274	(chronic adj2 (disease* or ill* or condition*)).tw,kw.
275	((medical or oral) adj4 condition*).tw,kw.
276	((dental plaque or dmf or gingival or periodontal) adj1 (index* or indices)).tw,kw.
277	(oral hygiene adj1 (index* or indices)).tw,kw.
278	(dentin* adj1 secondary).tw,kw.
279	(pulpitis or pulpitides).tw,kw.
280	(inflammation* adj1 endodontic).tw,kw.
281	(t??th adj1 (endodontically treated or nonvital or devitalized or pulpless)).tw,kw.
282	((sensitivit* or hypersensitivit*) adj1 (dentin or dentine or t??th)).tw,kw.
283	(((focal adj1 infection*) or fluoros?s or overjet) adj1 dental).tw,kw.
284	(mottled adj1 enamel*).tw,kw.
285	((t??th or dentoalveolar or dental) adj2 ankylos?s).tw,kw.
286	((decay or caries or fissure*) adj2 dental).tw,kw.
287	(caries adj2 stage*).tw,kw.
288	(white adj1 spot*).tw,kw.

289	(dental adj3 spot*).tw,kw.
290	(carious adj2 dentin*).tw,kw.
291	((cary or caries) adj2 (cervical or root)).tw,kw.
292	(odontalgia* or toothache* or tooth pain).tw,kw.
293	(disease* adj2 non communicable).tw,kw.
294	non infectious disease*.tw,kw.
295	(dental adj1 plaque).tw,kw.
296	sepsis.tw,kw.
297	((periodontal or periapical) adj1 disease*).tw,kw.
298	(disease* adj1 gingival).tw,kw.
299	(periodontiti* or periodontos?s or pericementitis or gingiviti* or fusospirillos?s or pericoroniti*).tw,kw.
300	(pocket* adj1 gingival).tw,kw.
301	(gingiv* adj2 (atroph* or recession*)).tw,kw.
302	((alveolar or periodont*) adj2 (bone loss* or atroph* or resorption)).tw,kw.
303	(periodontal attachment adj1 loss).tw,kw.
304	((periodontal or gingival) adj1 (abscess* or pocket*)).tw,kw.
305	suffering*.tw,kw.
306	((psychologic* or life or emotional or disorder* or management or mental or psychic or psycho social) adj1 stress*).tw,kw.
307	(emotional adj (factor or response or status or structure)).tw,kw.
308	emotion*.tw,kw.
309	((Mental or psychic) adj1 Tension).tw,kw.
310	schizotypal personality.tw,kw.
311	(personality adj1 (characteristic or pattern or structure or type)).tw,kw.
312	(burn out or burnout).tw,kw.
313	((work place or workplace or work or job or occupational or professional) adj2 stress*).tw,kw.
314	(saliva* or spittle).tw,kw.
315	(blood adj1 poisoning*).tw,kw.
316	(seps?s or septicemia* or bacteremia* or fungemia*).tw,kw.

317	((endotoxic or septic or toxic) adj1 shock).tw,kw.
318	((right or respect or sanctit* or value) adj2 life).tw,kw.
319	(shy* or timid* or assertiveness).tw,kw.
320	(family adj1 planning).tw,kw.
321	(family adj1 allowance*).tw,kw.
322	(family adj2 health).tw,kw.
323	(family adj5 life).tw,kw.
324	(family adj3 well*).tw,kw.
325	(family adj1 (dynamic* or relation* or interaction* or outcome* or histor*)).tw,kw.
326	((family or interparental or marital) adj1 conflict*).tw,kw.
327	(family adj2 relation*).tw,kw.
328	or/127-327
329	126 and 328
330	limit 329 to animals
331	329 not 330
332	limit 331 to (english language and yr="1999 -Current" and (article or article in press))
333	limit 332 to (evidence based medicine or consensus development or meta analysis or outcomes research or "systematic review" or "qualitative (best balance of sensitivity and specificity)")
334	Clinical study/ or Case control study/ or Family study/ or Longitudinal study/ or Retrospective study/ or (Prospective study/ not Randomized controlled trials/) or Cohort analysis/ or (Cohort adj (study or studies)).mp. or (Case control adj (study or studies)).tw. or (follow up adj (study or studies)).tw. or (observational adj (study or studies)).tw. or (epidemiologic$ adj (study or studies)).tw. or (cross sectional adj (study or studies)).tw.
335	332 and 334
336	333 or 335
337	(Equipment* or device* or supply or supplies or t??th brush* or t??thbrush or tooth paste or toothpaste or versus).ti.
338	(rats or rat or mice or dog* or pig* or horse* or dog* or mouse or rabbit* or animal*).tw,kw.
339	(dental adj2 (student* or school* or university or universities or college*)).tw,kw.
340	337 or 338 or 339
341	336 not 340

Key

/ = indexing term (Emtree heading)

exp = exploded indexing term (Emtree heading)

$ = truncation

tw = text word search in title or abstract fields

kw = terms in author provided keyword exact

pt = publication type.

adjn = terms within (n-1) words of each other (any order)

3. **CINAHL:**

Language: English

Age group: No limitation

Publication year: after 1999

Publication Types: Evidence-Based Care Sheet**,** Meta-analysis, Meta Synthesis, Systematic Reviews

Clinical Queries: Review (best balance of sensitivity and specificity), Qualitative: (best balance of sensitivity and specificity)

External filters used: qualitative studies, cohort studies, case control studies

Exclusion: equipment and supplies, all animal studies

Searched on November 19, 2021

Records retrieved: 7923

Interface - EBSCOhost Research Databases
Database - CINAHL Plus with Full Text
Search history sorted by search number descending.

S287	S282 NOT S286	Search modes – Boolean/Phrase
S286	S283 OR S284 OR S285	Search modes - Boolean/Phrase
S285	TI (dental N0 (student* or school* or university or universities or college*)) OR AB (dental N0 (student* or school* or university or universities or college*))	Search modes - Boolean/Phrase
S284	TI (Equipment* or instrument* or device* or supply or supplies or t??th brush* or t??thbrush or tooth paste or toothpaste or versus) OR AB (Equipment* or instrument* or device* or supply or supplies or t??th brush* or t??thbrush or tooth paste or toothpaste or versus)	Search modes - Boolean/Phrase
S283	TI (rats or rat or mice or dog* or pig or horse* or mouse or rabbit or animal*) OR AB (rats or rat or mice or dog* or pig or horse* or mouse or rabbit or animal*))	Search modes - Boolean/Phrase
S282	S275 OR S280 OR S281	Search modes - Boolean/Phrase
S281	S274	Limiters - Clinical Queries: Review - Best Balance, Qualitative - Best Balance<br>Search modes - Boolean/Phrase
S280	S274 AND S279	Search modes - Boolean/Phrase
S279	S276 OR S277 OR S278	Search modes - Boolean/Phrase
S278	(TI (interview)) OR (AB (interview)) OR (MH "Audiorecording") OR (TI ("qualitative stud*")) OR (AB ("qualitative stud*"))	Search modes - Boolean/Phrase

S277	(TI (cohort)) OR (AB (cohort)) OR (MH "Cohort Studies") OR (TI (longitudinal)) OR (AB (longitudinal)) OR (TI (prospective)) OR (AB (prospective)) OR (TI (retrospective)) OR (AB (retrospective))	Search modes - Boolean/Phrase
S276	(MH "Case Control Studies+") or (MH "Control Group") or (MH "Matched-Pair Analysis") or (TI (case or cases) n5 TI (control or controls)) OR (AB (case or cases) n5 AB (control or controls)) OR (TI (case or cases) n3 TI (matched)) OR (AB (case or cases) n3 AB (matched)) OR TI (control group*)	Search modes - Boolean/Phrase
S275	S274	Limiters - Publication Type: Evidence-Based Care Sheet, Meta Analysis, Meta Synthesis, Systematic Review<br>Search modes - Boolean/Phrase
S274	S273	Limiters - Published Date: 19990101-20190831; English Language<br>Search modes - Boolean/Phrase
S273	S137 AND S272	Search modes - Boolean/Phrase
S272	S138 OR S139 OR S140 OR S141 OR S142 OR S143 OR S144 OR S145 OR S146 OR S147 OR S148 OR S149 OR S150 OR S151 OR S152 OR S153 OR S154 OR S155 OR S156 OR S157 OR S158 OR S159 OR S160 OR S161 OR S162 OR S163 OR S164 OR S165 OR S166 OR S167 OR S168 OR S169 OR S170 OR S171 OR S172 OR S173 OR S174 OR S175 OR S176 OR S177 OR S178 OR S179 OR S180 OR S181 OR S182 OR S183 OR S184 OR S185 OR S186 OR S187 OR S188 OR S189 OR S190 OR S191 OR S192 OR S193 OR S194 OR S195 OR S196 OR S197 OR S198 OR S199 OR S200 OR S201 OR S202 OR S203 OR S204 OR S205 OR S206 OR S207 OR S208 OR S209 OR S210 OR S211	Search modes - Boolean/Phrase

	OR S212 OR S213 OR S214 OR S215 OR S216 OR S217 OR S218 OR S219 OR S220 OR S221 OR S222 OR S223 OR S224 OR S225 OR S226 OR S227 OR S228 OR S229 OR S230 OR S231 OR S232 OR S233 OR S234 OR S235 OR S236 OR S237 OR S238 OR S239 OR S240 OR S241 OR S242 OR S243 OR S244 OR S245 OR S246 OR S247 OR S248 OR S249 OR S250 OR S251 OR S252 OR S253 OR S254 OR S255 OR S256 OR S257 OR S258 OR S259 OR S260 OR S261 OR S262 OR S263 OR S264 OR S265 OR S266 OR S267 OR S268 OR S269 OR S270 OR S271	
S271	TI health N0 status OR AB health N0 status	Search modes - Boolean/Phrase
S270	(MH "Health Status") OR (MH "Health Status Disparities")	Search modes - Boolean/Phrase
S269	TI (absenteeism or presenteeism) OR AB (absenteeism or presenteeism)	Search modes - Boolean/Phrase
S268	(MH "Absenteeism") OR (MH "Presenteeism")	Search modes - Boolean/Phrase
S267	TI (expenditure* or expense*) OR AB (expenditure* or expense*)	Search modes - Boolean/Phrase
S266	TI (“out of pocket” N0 (cost* or pay* or spending)) OR AB (“out of pocket” N0 (cost* or pay* or spending))	Search modes - Boolean/Phrase
S265	TI (cost* N0 (effectiveness or comparison* or measure* or allocation* or apportionment* or shifting* or containment* or control* or sharing or saving*)) OR AB (cost* N0 (effectiveness or comparison* or measure* or allocation* or apportionment* or shifting* or containment* or control* or sharing or saving*))	Search modes - Boolean/Phrase
S264	TI (cost* N1 (analys?s or benefit*)) OR AB (cost* N1 (analys?s or benefit*))	Search modes - Boolean/Phrase
S263	TI marginal N0 analys?s OR AB marginal N0 analys?s	Search modes - Boolean/Phrase
S262	TI expropriation* OR AB expropriation*	Search modes - Boolean/Phrase
S261	TI ((healthcare or “health care”) N0 ration*) OR AB ((healthcare or “health care”) N0 ration*)	Search modes - Boolean/Phrase

S260	TI allocative N0 efficiency OR AB allocative N0 efficiency	Search modes - Boolean/Phrase
S259	TI resource N1 allocation* OR AB resource N1 allocation*	Search modes - Boolean/Phrase
S258	TI compensation* OR AB compensation*	Search modes - Boolean/Phrase
S257	TI utility N0 theor* OR AB utility N0 theor*	Search modes - Boolean/Phrase
S256	TI productivity OR AB productivity	Search modes - Boolean/Phrase
S255	TI “household consumption*” OR AB “household consumption*”	Search modes - Boolean/Phrase
S254	TI easterlin N0 hypothesis OR AB easterlin N0 hypothesis	Search modes - Boolean/Phrase
S253	TI cost* N1 living OR AB cost* N1 living	Search modes - Boolean/Phrase
S252	TI Consumption OR AB Consumption	Search modes - Boolean/Phrase
S251	TI (“consumer price” N0 (index* or indices)) OR AB (“consumer price” N0 (index* or indices))	Search modes - Boolean/Phrase
S250	TI literac* OR AB literac*	Search modes - Boolean/Phrase
S249	TI educational N1 status* OR AB educational N1 status*	Search modes - Boolean/Phrase
S248	TI ((high* or middle or low* or develop*) N1 countr*) OR AB ((high* or middle or low* or develop*) N1 countr*)	Search modes - Boolean/Phrase
S247	TI (income N1 (group* or level* or classification* or high* or middle or low*)) OR AB (income N1 (group* or level* or classification* or high* or middle or low*))	Search modes - Boolean/Phrase
S246	TI ((macroeconomic or microeconomic or socioeconomic) N0 (factor* or aspect*)) OR AB ((macroeconomic or microeconomic or socioeconomic) N0 (factor* or aspect*))	Search modes - Boolean/Phrase

S245	TI economic* OR AB economic*	Search modes - Boolean/Phrase
S244	TI ((hospital or patient) N0 readmission*) OR AB ((hospital or patient) N0 readmission*)	Search modes - Boolean/Phrase
S243	TI ((patient or voluntary) N0 admission*) OR AB ((patient or voluntary) N0 admission*)	Search modes - Boolean/Phrase
S242	TI length* N1 stay* OR AB length* N1 stay*	Search modes - Boolean/Phrase
S241	TI hospital stay* OR AB hospital stay*	Search modes - Boolean/Phrase
S240	TI hospital N1 service* OR AB hospital N1 service*	Search modes - Boolean/Phrase
S239	TI hospitali?ation* OR AB hospitali?ation*	Search modes - Boolean/Phrase
S238	TI health N1 manpower OR AB health N1 manpower	Search modes - Boolean/Phrase
S237	TI (health N0 (resource* or workforce)) OR AB (health N0 (resource* or workforce))	Search modes - Boolean/Phrase
S236	TI ((back or return) N1 work) OR AB ((back or return) N1 work)	Search modes - Boolean/Phrase
S235	TI career N0 mobilit* OR AB (career N0 mobilit*	Search modes - Boolean/Phrase
S234	TI ((career or clinical or job) N0 ladder*) OR AB ((career or clinical or job) N0 ladder*)	Search modes - Boolean/Phrase
S233	TI underemployment OR AB underemployment	Search modes - Boolean/Phrase
S232	TI occupational N1 status OR AU occupational N1 status	Search modes - Boolean/Phrase
S231	TI labo#r force OR AB labo#r force	Search modes - Boolean/Phrase

S230	TI employment OR AB employment	Search modes - Boolean/Phrase
S229	TI ((work or job or vocation* or life) N2 (satisfaction* or performance* or stress*)) OR AB ((work or job or vocation* or life) N2 (satisfaction* or performance* or stress*))	Search modes - Boolean/Phrase
S228	TI "Uncompensated care" OR AB "Uncompensated care"	Search modes - Boolean/Phrase
S227	TI ((senior or aged or elderly) N2 (centre* or center* or home* or facilit*)) OR AB ((senior or aged or elderly) N2 (centre* or center* or home* or facilit*))	Search modes - Boolean/Phrase
S226	TI (("displaced person*" or refugee) N0 (camp* or settlement* or shelter*)) OR AB (("displaced person*" or refugee) N0 (camp* or settlement* or shelter*))	Search modes - Boolean/Phrase
S225	TI ((emergency or evacuation) N0 shelter*) OR AB ((emergency or evacuation) N0 shelter*)	Search modes - Boolean/Phrase
S224	TI ((employment or occupational or employee*) N2 (health or assistance)) OR AB ((employment or occupational or employee*) N2 (health or assistance))	Search modes - Boolean/Phrase
S223	TI Maternal N0 health N0 service* OR AB Maternal N0 health N0 service*	Search modes - Boolean/Phrase
S222	TI Hospice* OR AB Hospice*	Search modes - Boolean/Phrase
S221	TI ((home or domiciliary) N0 care) OR AB ((home or domiciliary) N0 care)	Search modes - Boolean/Phrase
S220	TI ((foster or kinship) N1 care) OR AB ((foster or kinship) N1 care)	Search modes - Boolean/Phrase
S219	TI (planned N0 (pregnanc* or parenthood)) OR AB (planned N0 (pregnanc* or parenthood))	Search modes - Boolean/Phrase
S218	TI family N0 planning OR AB family N0 planning	Search modes - Boolean/Phrase
S217	TI community N0 pharmac* N0 service* OR AB community N0 pharmac* N0 service*	Search modes - Boolean/Phrase
S216	TI community N0 network* OR AB community N0 network*	Search modes - Boolean/Phrase

S215	TI assertive N0 community N0 treatment* OR AB assertive N0 community N0 treatment*	Search modes - Boolean/Phrase
S214	TI ((“public health” or community) N1 nursing) OR AB ((“public health” or community) N1 nursing)	Search modes - Boolean/Phrase
S213	TI (daycare N0 (center* or centre*)) OR AB (daycare N0 (center* or centre*))	Search modes - Boolean/Phrase
S212	TI (community N0 (integration* or reintegration* or program*)) OR AB (community N0 (integration* or reintegration* or program*))	Search modes - Boolean/Phrase
S211	TI (community N1 (health or care)) OR AB (community N1 (health or care))	Search modes - Boolean/Phrase
S210	TI (functional N0 (assessment* or training*)) OR AB (functional N0 (assessment* or training*))	Search modes - Boolean/Phrase
S209	TI (rehabilitation* or readaptation* or readjustment* or recover* or resociali?ation* or revalidation*) OR AB (rehabilitation* or readaptation* or readjustment* or recover* or resociali?ation* or revalidation*)	Search modes - Boolean/Phrase
S208	TI medical N1 overus* OR AB medical N1 overus*	Search modes - Boolean/Phrase
S207	TI ((preventative or physician* or diagnostic) N1 service*) OR AB ((preventative or physician* or diagnostic) N1 service*)	Search modes - Boolean/Phrase
S206	TI (health N1 (service* or agenc* or practice*)) OR AB (health N1 (service* or agenc* or practice*))	Search modes - Boolean/Phrase
S205	TI health N2 system* OR AB health N2 system*	Search modes - Boolean/Phrase
S204	TI ((drug or medicine) N1 (us* or misus* or consumption*)) OR AB ((drug or medicine) N1 (us* or misus* or consumption*))	Search modes - Boolean/Phrase
S203	TI drug N2 utilization OR AB drug N2 utilization	Search modes - Boolean/Phrase
S202	TI responsiveness OR AB responsiveness	Search modes - Boolean/Phrase
S201	TI time N3 loss OR AB time N3 loss	Search modes - Boolean/Phrase

S200	TI health N2 indicator* N2 indicator* OR AB health N2 indicator*	Search modes - Boolean/Phrase
S199	TI organizational N0 involvement* OR AB organizational N0 involvement*	Search modes - Boolean/Phrase
S198	TI triage* OR AB triage*	Search modes - Boolean/Phrase
S197	TI patient* N1 transport* OR AB patient* N1 transport*	Search modes - Boolean/Phrase
S196	TI emergency N0 psychiatric N1 service* OR AB emergency N0 psychiatric N1 service*	Search modes - Boolean/Phrase
S195	TI (emergency N2 (visit* or admission*)) OR AB (emergency N2 (visit* or admission*))	Search modes - Boolean/Phrase
S194	TI emergency N1 unit OR AB emergency N1 unit	Search modes - Boolean/Phrase
S193	TI (trauma N0 (center* or centre*)) OR AB (trauma N0 (center* or centre*))	Search modes - Boolean/Phrase
S192	TI (emergency N0 (ward* or department* or room* or centre* or center* or dispensar*)) OR AB (emergency N0 (ward* or department* or room* or centre* or center* or dispensar*))	Search modes - Boolean/Phrase
S191	TI emergency N0 hospital N0 service* OR AB emergency N0 hospital N0 service*	Search modes - Boolean/Phrase
S190	TI emergency N1 dispatch* OR AB emergency N1 dispatch*	Search modes - Boolean/Phrase
S189	TI “advanced trauma life support” OR AB “advanced trauma life support”	Search modes - Boolean/Phrase
S188	TI emergicenter* OR AB emergicenter*	Search modes - Boolean/Phrase
S187	TI emergency N1 care OR AB emergency N1 care	Search modes - Boolean/Phrase
S186	TI ((accident* or emergenc*) N1 service*) OR AB ((accident* or emergenc*) N1 service*)	Search modes - Boolean/Phrase

S185	TI productivity OR AB productivity	Search modes - Boolean/Phrase
S184	TI efficiency OR AB efficiency	Search modes - Boolean/Phrase
S183	TI (sport* or athletic*) OR AB (sport* or athletic*)	Search modes - Boolean/Phrase
S182	TI (hobby or hobbies) OR AB (hobby or hobbies)	Search modes - Boolean/Phrase
S181	TI relaxation* OR AB relaxation*	Search modes - Boolean/Phrase
S180	TI recreation* OR AB recreation*	Search modes - Boolean/Phrase
S179	TI (festival* or holiday*) OR AB (festival* or holiday*)	Search modes - Boolean/Phrase
S178	TI leisure* OR AB leisure*	Search modes - Boolean/Phrase
S177	TI ((psychological or psychosocial or social) N0 support*) OR AB ((psychological or psychosocial or social) N0 support*)	Search modes - Boolean/Phrase
S176	TI family N0 allowance* OR AB family N0 allowance*	Search modes - Boolean/Phrase
S175	TI aid N2 “families with dependent children” OR AB aid N2 “families with dependent children”	Search modes - Boolean/Phrase
S174	TI (social N0 (security or insurance)) OR AB (social N0 (security or insurance))	Search modes - Boolean/Phrase
S173	TI “social welfare” OR AB “social welfare”	Search modes - Boolean/Phrase
S172	TI federal N1 aid* OR AB federal N1 aid*	Search modes - Boolean/Phrase
S171	TI investment* OR AB investment*	Search modes - Boolean/Phrase

S170	TI ((healthcare or “health care”) N0 (industr* or market* or sector*)) OR AB ((healthcare or “health care”) N0 (industr* or market* or sector*))	Search modes - Boolean/Phrase
S169	TI health N1 marketing OR AB health N1 marketing	Search modes - Boolean/Phrase
S168	TI ((group or shared or joint) N0 purchasing) OR AB ((group or shared or joint) N0 purchasing)	Search modes - Boolean/Phrase
S167	TI "direct service" N0 cost* OR AB "direct service" N0 cost*	Search modes - Boolean/Phrase
S166	TI (charge* or fee or fees) OR AB (charge* or fee or fees)	Search modes - Boolean/Phrase
S165	TI debt* OR AB debt*	Search modes - Boolean/Phrase
S164	TI (medical or health* or treatment or drug or hospital) N1 cost*) OR AB (medical or health* or treatment or drug or hospital) N1 cost*)	Search modes - Boolean/Phrase
S163	TI pricing OR AB pricing	Search modes - Boolean/Phrase
S162	TI affordabilit* OR AB affordabilit*	Search modes - Boolean/Phrase
S161	TI "return on investment" OR AB "return on investment" OR TI ROI OR AB ROI	Search modes - Boolean/Phrase
S160	(MH "Productivity")	Search modes - Boolean/Phrase
S159	(MH "Leisure Activities+")	Search modes - Boolean/Phrase
S158	(MH "Support, Psychosocial")	Search modes - Boolean/Phrase
S157	(MH "Economics+")	Search modes - Boolean/Phrase
S156	(MH "Work Environment")	Search modes - Boolean/Phrase

S155	(MH "Downsizing, Organizational")	Search modes - Boolean/Phrase
S154	(MH "Job Re-Entry")	Search modes - Boolean/Phrase
S153	(MH "Career Mobility+")	Search modes - Boolean/Phrase
S152	(MH "Employment+") OR (MH "Unemployment")	Search modes - Boolean/Phrase
S151	(MH "Job Satisfaction")	Search modes - Boolean/Phrase
S150	(MH "Role") OR (MH "Professional Role") OR (MH "Nursing Role") OR (MH "Physician's Role") OR (MH "Sick Role")	Search modes - Boolean/Phrase
S149	(MH "Self Care") OR (MH "Self Care Agency") OR (MH "Self-Management")	Search modes - Boolean/Phrase
S148	(MH "Activities of Daily Living") OR (MH "Social Participation")	Search modes - Boolean/Phrase
S147	(MH "Rehabilitation")	Search modes - Boolean/Phrase
S146	(MH "Poison Control Centers")	Search modes - Boolean/Phrase
S145	(MH "Advanced Trauma Life Support Care")	Search modes - Boolean/Phrase
S144	(MH "Emergency Medical Services+")	Search modes - Boolean/Phrase
S143	(MH "Hospitalization+") OR (MH "Length of Stay") OR (MH "Patient Admission") OR (MH "Patient Discharge") OR (MH "Patient Dumping") OR (MH "Readmission")	Search modes - Boolean/Phrase
S142	(MH "Hospices")	Search modes - Boolean/Phrase
S141	(MH "Child Health Services+")	Search modes - Boolean/Phrase

S140	(MH "Community Health Services+")	Search modes - Boolean/Phrase
S139	(MH "Health Services")	Search modes - Boolean/Phrase
S138	(MH "Drug Utilization+") OR (MH "Utilization Review")	Search modes - Boolean/Phrase
S137	S26 OR S136	Search modes - Boolean/Phrase
S136	S125 OR S135	Search modes - Boolean/Phrase
S135	S133 OR S134	Search modes - Boolean/Phrase
S134	S131	Limiters - Special Interest: Dental Care<br>Search modes - Boolean/Phrase
S133	S131 AND S132	Search modes - Boolean/Phrase
S132	(MH "Dentistry")	Search modes - Boolean/Phrase
S131	S126 OR S127 OR S128 OR S129 OR S130	Search modes - Boolean/Phrase
S130	TI ((public or deliver* or system*) N0 ("health care" or healthcare)) OR AB ((public or deliver* or system*) N0 ("health care" or healthcare))	Search modes - Boolean/Phrase
S129	TI (Pathology or pathological or telepathology) OR AB (Pathology or pathological or telepathology)	Search modes - Boolean/Phrase
S128	TI "head start" N0 program* OR AB "head start" N0 program*	Search modes - Boolean/Phrase
S127	TI program* N1 wellness OR AB program* N1 wellness	Search modes - Boolean/Phrase

S126	TI ((promotion* or campaign* or program*) N1 health) OR AB ((promotion* or campaign* or program*) N1 health)	Search modes - Boolean/Phrase
S125	S27 OR S28 OR S29 OR S30 OR S31 OR S32 OR S33 OR S34 OR S35 OR S36 OR S37 OR S38 OR S39 OR S40 OR S41 OR S42 OR S43 OR S44 OR S45 OR S46 OR S47 OR S48 OR S49 OR S50 OR S51 OR S52 OR S53 OR S54 OR S55 OR S56 OR S57 OR S58 OR S59 OR S60 OR S61 OR S62 OR S63 OR S64 OR S65 OR S66 OR S67 OR S68 OR S69 OR S70 OR S71 OR S72 OR S73 OR S74 OR S75 OR S76 OR S77 OR S78 OR S79 OR S80 OR S81 OR S82 OR S83 OR S84 OR S85 OR S86 OR S87 OR S88 OR S89 OR S90 OR S91 OR S92 OR S93 OR S94 OR S95 OR S96 OR S97 OR S98 OR S99 OR S100 OR S101 OR S102 OR S103 OR S104 OR S105 OR S106 OR S107 OR S108 OR S109 OR S110 OR S111 OR S112 OR S113 OR S114 OR S115 OR S116 OR S117 OR S118 OR S119 OR S120 OR S121 OR S122 OR S123 OR S124	Search modes - Boolean/Phrase
S124	TI medicine N0 oral OR AB medicine N0 oral	Search modes - Boolean/Phrase
S123	TI stomatolog* OR AB stomatolog*	Search modes - Boolean/Phrase
S122	TI (mouth N0 protect*) OR AB (mouth N0 protect*)	Search modes - Boolean/Phrase
S121	TI (mouth N0 guard) OR AB (mouth N0 guard)	Search modes - Boolean/Phrase
S120	TI fluoridation* OR AB fluoridation*	Search modes - Boolean/Phrase
S119	TI ((subgingival or gingival) N0 (curettage* or retraction*)) OR AB ((subgingival or gingival) N0 (curettage* or retraction*))	Search modes - Boolean/Phrase
S118	TI root N1 planing* OR AB root N1 planing*	Search modes - Boolean/Phrase
S117	TI ((dental or mouth or t??th) N0 debridement*) OR AB ((dental or mouth or t??th) N0 debridement*)	Search modes - Boolean/Phrase
S116	TI (periodontal N1 (“tissue regeneration*” or debridement*)) OR AB (periodontal N1 (“tissue regeneration*” or debridement*))	Search modes - Boolean/Phrase
S115	TI (periodontal N0 (medicine*or prosthes?s or splint* or dressing* or prevention*)) OR AB (periodontal N0 (medicine*or prosthes?s or splint* or dressing* or prevention*))	Search modes - Boolean/Phrase

S114	TI ((oral or maxillofacial) N2 patholog*) OR AB ((oral or maxillofacial) N2 patholog*)	Search modes - Boolean/Phrase
S113	TI (tooth N1 (movement* or intrusion* or depression* or care)) OR AB (tooth N1 (movement* or intrusion* or depression* or care))	Search modes - Boolean/Phrase
S112	TI (palate N0 (plast* or reconstruction* or operation*)) OR AB (palate N0 (plast* or reconstruction* or operation*))	Search modes - Boolean/Phrase
S111	TI palatoplast* OR AB palatoplast*	Search modes - Boolean/Phrase
S110	TI ((maxillary or palatal) N0 expansion*) OR AB ((maxillary or palatal) N0 expansion*)	Search modes - Boolean/Phrase
S109	TI forced N1 eruption* OR AB forced N1 eruption*	Search modes - Boolean/Phrase
S108	TI crown N0 lengthening OR AB crown N0 lengthening	Search modes - Boolean/Phrase
S107	TI invisalign* OR AB invisalign*	Search modes - Boolean/Phrase
S106	TI bionator* OR AB bionator*	Search modes - Boolean/Phrase
S105	TI jasper N0 jumper* OR AB jasper N0 jumper*	Search modes - Boolean/Phrase
S104	TI ((function or harvold) N0 activator*) OR AB ((function or harvold) N0 activator*)	Search modes - Boolean/Phrase
S103	TI frankel N1 regulator* OR AB frankel N1 regulator*	Search modes - Boolean/Phrase
S102	TI ((clear aligner or herbst or bimler or andresen) N0 appliance*) OR AB ((clear aligner or herbst or bimler or andresen) N0 appliance*)	Search modes - Boolean/Phrase
S101	TI ((fixed or bonded or permanent) N0 retainer*) OR AB ((fixed or bonded or permanent) N0 retainer*)	Search modes - Boolean/Phrase
S100	TI ((fixed or activator or extraoral) N1 appliance*) OR AB ((fixed or activator or extraoral) N1 appliance*)	Search modes - Boolean/Phrase

S99	TI (occlusal N0 (splint* or adjustment* or equilibration*)) OR AB (occlusal N0 (splint* or adjustment* or equilibration*))	Search modes - Boolean/Phrase
S98	TI mandibular N0 advancement OR AB mandibular N0 advancement	Search modes - Boolean/Phrase
S97	TI (dental N0 (internal or marginal) N0 adaptation*) OR AB (dental N0 (internal or marginal) N0 adaptation*)	Search modes - Boolean/Phrase
S96	TI (uvulopharyngopalatoplast* or uvulopalatoplast* or palatouvulopharyngoplast* or pharyngouvulopalatoplast*) OR AB (uvulopharyngopalatoplast* or uvulopalatoplast* or palatouvulopharyngoplast* or pharyngouvulopalatoplast*)	Search modes - Boolean/Phrase
S95	TI odontectom* OR AB odontectom*	Search modes - Boolean/Phrase
S94	TI (Molar N0 (amputation* or extraction*)) OR AB (Molar N0 (amputation* or extraction*))	Search modes - Boolean/Phrase
S93	TI Exodont* OR AB Exodont*	Search modes - Boolean/Phrase
S92	TI ((t??th or serial) N0 extraction*) OR AB ((t??th or serial) N0 extraction*)	Search modes - Boolean/Phrase
S91	TI sinus N1 augmentation* OR AB sinus N1 augmentation*	Search modes - Boolean/Phrase
S90	TI “sagittal split” N1 osteotom* OR AB “sagittal split” N1 osteotom*	Search modes - Boolean/Phrase
S89	TI “Le fort” N0 operation* OR AB “Le fort” N0 operation*	Search modes - Boolean/Phrase
S88	TI ((“le fort” or lefort or maxilla* or jaw or mandib*) N1 osteotom*) OR AB ((“le fort” or lefort or maxilla* or jaw or mandib*) N1 osteotom*)	Search modes - Boolean/Phrase
S87	TI alveolar N1 graft* OR AB alveolar N1 graft*	Search modes - Boolean/Phrase
S86	TI ((endosseous or subperiosteal) N1 implant*) OR AB ((endosseous or subperiosteal) N1 implant*)	Search modes - Boolean/Phrase
S85	TI (alveolectom* or alveoloplast* or vestibuloplast*) OR AB (alveolectom* or alveoloplast* or vestibuloplast*)	Search modes - Boolean/Phrase

S84	TI ((maxillary or mandibular or alveolar) N0 ridge augmentation*) OR AB ((maxillary or mandibular or alveolar) N0 ridge augmentation*)	Search modes - Boolean/Phrase
S83	TI (chinplast* or mentoplast*) OR AB (chinplast* or mentoplast*)	Search modes - Boolean/Phrase
S82	TI (chin N1 (correction* or reconstruction * or reduction* or surger* or operation*)) OR AB (chin N1 (correction* or reconstruction * or reduction* or surger* or operation*))	Search modes - Boolean/Phrase
S81	TI genioplast* OR AB genioplast*	Search modes - Boolean/Phrase
S80	TI maxillectom* OR AB maxillectom*	Search modes - Boolean/Phrase
S79	TI ((upper jaw or maxilla*) N0 resection)) OR AB ((upper jaw or maxilla*) N0 resection))	Search modes - Boolean/Phrase
S78	TI ((maxillofacial or mandibular) N0 (prosthes?s or impant*)) OR AB ((maxillofacial or mandibular) N0 (prosthes?s or impant*))	Search modes - Boolean/Phrase
S77	TI (“mandible ostectom*” or mandibulectom*) OR AB (“mandible ostectom*” or mandibulectom*)	Search modes - Boolean/Phrase
S76	TI (mandibula* N0 (advancement* or reconstruction* or restoration* or resection*)) OR AB (mandibula* N0 (advancement* or reconstruction* or restoration* or resection*))	Search modes - Boolean/Phrase
S75	TI ((jaw or maxillomandibular) N0 fixation*) OR AB ((jaw or maxillomandibular) N0 fixation*)	Search modes - Boolean/Phrase
S74	TI ((tongue or lingual) N0 (extirpation* or resection*)) OR AB ((tongue or lingual) N0 (extirpation* or resection*))	Search modes - Boolean/Phrase
S73	TI (apicoectom* or gingivectom* or gingivoplast* or glossectom*) OR AB (apicoectom* or gingivectom* or gingivoplast* or glossectom*)	Search modes - Boolean/Phrase
S72	TI dental N0 infection N0 control* OR AB dental N0 infection N0 control*	Search modes - Boolean/Phrase
S71	TI (Dental N0 (reimplantation* or replantation* or reinclusion*)) OR AB (Dental N0 (reimplantation* or replantation* or reinclusion*))	Search modes - Boolean/Phrase
S70	TI (t??th N0 (bleaching or whitening or replantation* or reimplantation* or extrusion* or uprighting* or remineralization* or polishing or restoration* or surger* or inlay* or preparation* or removal* or resection*))) OR AB (t??th N0 (bleaching or whitening or replantation* or	Search modes - Boolean/Phrase

	reimplantation* or extrusion* or uprighting* or remineralization* or polishing or restoration* or surger* or inlay* or preparation* or removal* or resection*)))	
S69	TI ((dental or enamel) N0 microabrasion*) OR AB ((dental or enamel) N0 microabrasion*)	Search modes - Boolean/Phrase
S68	TI (dental N1 (implant* or prosthes?s)) OR AB (dental N1 (implant* or prosthes?s))	Search modes - Boolean/Phrase
S67	TI (Sinus N0 lifting*) OR AB (Sinus N0 or lifting*)	Search modes - Boolean/Phrase
S66	TI "Jaw relation record*" OR AB "Jaw relation record*"	Search modes - Boolean/Phrase
S65	TI ((tooth or dental) N0 (an#esthesia or an#esthetic* or casting*)) OR AB ((tooth or dental) N0 (an#esthesia or an#esthetic* or casting*))	Search modes - Boolean/Phrase
S64	TI ((dental or tooth or caries) N1 prevention) OR AB ((dental or tooth or caries) N1 prevention)	Search modes - Boolean/Phrase
S63	TI ((dental or tooth) N2 filling*) OR AB ((dental or tooth) N2 filling*)	Search modes - Boolean/Phrase
S62	TI (dental N0 (esthetic* or aesthetic* or floss or scaling* or brace* or hygiene* or polishing or radiology or bonding or soldering* or prosthetic*)) OR AB (dental N0 (esthetic* or aesthetic* or floss or scaling* or brace* or hygiene* or polishing or radiology or bonding or soldering* or prosthetic*))	Search modes - Boolean/Phrase
S61	TI ((evidence-based or preventative or cosmetic or operative or prosthetic or reparative) N1 dentistry) OR AB ((evidence-based or preventative or cosmetic or operative or prosthetic or reparative) N1 dentistry)	Search modes - Boolean/Phrase
S60	TI oral N0 hygiene OR AB oral N0 hygiene	Search modes - Boolean/Phrase
S59	TI ((maxillo-mandibular or maxillomandibular or oral or maxillofacial or orthognathic) N1 procedure*) OR AB ((maxillo-mandibular or maxillomandibular or oral or maxillofacial or orthognathic) N1 procedure*)	Search modes - Boolean/Phrase
S58	TI ((maxillo-mandibular or maxillomandibular or oral or maxillofacial or orthognathic or jaw) N1 surg*) OR AB ((maxillo-mandibular or maxillomandibular or oral or maxillofacial or orthognathic or jaw) N1 surg*)	Search modes - Boolean/Phrase

S57	TI ((supragingival or subgingival or dental or root) N0 scaling*) OR AB ((supragingival or subgingival or dental or root) N0 scaling*)	Search modes - Boolean/Phrase
S56	TI ((dental or periodontal or t??th) N0 prophylaxis) OR AB ((dental or periodontal or t??th) N0 prophylaxis)	Search modes - Boolean/Phrase
S55	TI community N0 dentistry OR AB community N0 dentistry	Search modes - Boolean/Phrase
S54	TI fluorescence N0 “quantitative light-induced” OR AB fluorescence N0 “quantitative light-induced”	Search modes - Boolean/Phrase
S53	TI (Dental N0 (“x ray” or xray)) OR AB (Dental N0 (“x ray” or xray))	Search modes - Boolean/Phrase
S52	TI ((dental or bitewing or tooth) N0 (radiography or radiovisiography)) OR AB ((dental or bitewing or tooth) N0 (radiography or radiovisiography))	Search modes - Boolean/Phrase
S51	TI ((dental or intraoral or orthodontic) N0 photograph*) OR AB ((dental or intraoral or orthodontic) N0 photograph*)	Search modes - Boolean/Phrase
S50	TI dental N0 pulp test* OR AB dental N0 pulp test*	Search modes - Boolean/Phrase
S49	TI ((diagnos* or examination*) N0 (oral or dental)) OR AB ((diagnos* or examination*) N0 (oral or dental))	Search modes - Boolean/Phrase
S48	TI ((mouth or oral) N1 rehabilitation*) OR AB ((mouth or oral) N1 rehabilitation*)	Search modes - Boolean/Phrase
S47	TI (“community periodontal index of treatment needs” or cpitn) OR AB (“community periodontal index of treatment needs” or cpitn)	Search modes - Boolean/Phrase
S46	TI varnish* N1 cavity OR AB varnish* N1 cavity	Search modes - Boolean/Phrase
S45	TI “cavity lining*” N0 dental OR AB “cavity lining*” N0 dental	Search modes - Boolean/Phrase
S44	TI ((“pre prosthetic” or preprosthetic) N0 surger*) OR AB ((“pre prosthetic” or preprosthetic) N0 surger*)	Search modes - Boolean/Phrase
S43	TI parotidectom* OR AB parotidectom*	Search modes - Boolean/Phrase

S42	TI ((oral or mouth) N1 surg*) OR AB ((oral or mouth) N1 surg*)	Search modes - Boolean/Phrase
S41	TI (root canal N0 (therap* or procedure* or obturation* or preparation* or surger*)) OR AB (root canal N0 (therap* or procedure* or obturation* or preparation* or surger*))	Search modes - Boolean/Phrase
S40	TI (Orthodontic N1 (“space closure” or anchorage* or extrusion*)) OR AB (Orthodontic N1 (“space closure” or anchorage* or extrusion*))	Search modes - Boolean/Phrase
S39	TI (orthodontia or orthodontolog* or orthodonty) OR AB (orthodontia or orthodontolog* or orthodonty)	Search modes - Boolean/Phrase
S38	TI (endodontics or orthodontics or periodontics or prosthodontics) OR AB (endodontics or orthodontics or periodontics or prosthodontics)	Search modes - Boolean/Phrase
S37	TI ((dental or t??th or periodontal) N0 cleaning) OR AB ((dental or t??th or periodontal) N0 cleaning)	Search modes - Boolean/Phrase
S36	TI ((periodont* or orthodontic* or prosthodont* or endodontic) N1 (treatment* or procedure* or surger* or care)) OR AB ((periodont* or orthodontic* or prosthodont* or endodontic) N1 (treatment* or procedure* or surger* or care))	Search modes - Boolean/Phrase
S35	TI “bleeding on probing” N2 gingival OR AB “bleeding on probing” N2 gingival	Search modes - Boolean/Phrase
S34	TI ((decayed or missing or filled) N2 teeth) OR AB ((decayed or missing or filled) N2 teeth)	Search modes - Boolean/Phrase
S33	TI “dental health” N1 survey* OR AB “dental health” N1 survey*	Search modes - Boolean/Phrase
S32	TI oral N1 care OR AB oral N1 care	Search modes - Boolean/Phrase
S31	TI denture* OR AB denture*	Search modes - Boolean/Phrase
S30	TI dental stress analys?s OR AB dental stress analys?s	Search modes - Boolean/Phrase
S29	TI (dent* N1 (“check-up” or health or intervention* or clinic* or treatment* or therap* or program* or practice* or education or procedure* or restorat* or regenerative*)) OR AB (dent* N1 (“check-up” or health or intervention* or clinic* or treatment* or therap* or program* or practice* or education or procedure* or restorat* or regenerative*))	Search modes - Boolean/Phrase

S28	TI dental N2 service* OR AB dental N2 service*	Search modes - Boolean/Phrase
S27	TI care N2 dental OR AB care N2 dental	Search modes - Boolean/Phrase
S26	S17 OR S25	Search modes - Boolean/Phrase
S25	S23 OR S24	Search modes - Boolean/Phrase
S24	S22	Limiters - Special Interest: Dental Care<br>Search modes - Boolean/Phrase
S23	S21 AND S22	Search modes - Boolean/Phrase
S22	S18 OR S19 OR S20	Search modes - Boolean/Phrase
S21	(MH "Dentistry+")	Search modes - Boolean/Phrase
S20	(MH "Patient Education+")	Search modes - Boolean/Phrase
S19	(MH "Public Health")	Search modes - Boolean/Phrase
S18	(MH "Health Promotion+")	Search modes - Boolean/Phrase
S17	S1 OR S2 OR S3 OR S4 OR S5 OR S6 OR S7 OR S8 OR S9 OR S10 OR S11 OR S12 OR S13 OR S14 OR S15 OR S16	Search modes - Boolean/Phrase
S16	(MH "Oral Medicine")	Search modes - Boolean/Phrase
S15	(MH "Prosthodontics+")	Search modes - Boolean/Phrase

S14	(MH "Preventive Dentistry+")	Search modes - Boolean/Phrase
S13	(MH "Periodontics+")	Search modes - Boolean/Phrase
S12	(MH "Pathology, Oral")	Search modes - Boolean/Phrase
S11	(MH "Orthodontics+")	Search modes - Boolean/Phrase
S10	(MH "Surgery, Oral+")	Search modes - Boolean/Phrase
S9	(MH "Esthetics, Dental")	Search modes - Boolean/Phrase
S8	(MH "Endodontics+")	Search modes - Boolean/Phrase
S7	(MH "Dentistry, Operative+")	Search modes - Boolean/Phrase
S6	(MH "Dental Health Services+")	Search modes - Boolean/Phrase
S5	(MH "Dental Prophylaxis+")	Search modes - Boolean/Phrase
S4	(MH "Diagnosis, Oral+")	Search modes - Boolean/Phrase
S3	(MH "Public Health Dentistry") OR (MH "Dental Health Education")	Search modes - Boolean/Phrase
S2	(MH "Dental Clinics")	Search modes - Boolean/Phrase
S1	(MH "Dental Care+")	Search modes - Boolean/Phrase

Key

MH = indexing term (CINAHL heading)

* = truncation

TI = terms in the title

AB = terms in the abstract

“ “ = phrase search.

Nn = terms within n words of each other (any order)

# = Optional wildcards (stands for 1 or 0 characters)

? = Mandatory wildcards (stands for exactly 1 character)

4. **Sociological Abstracts**

Language: English

Publication year: after 1999

Searched on Aug.27, 2019

Records retrieved: 372

Search Strategy:

Since this is a very specific database, we only searched the terms for the "Intervention" and did not search the terms for the "outcome."

su(dental care) OR ((noft((care NEAR/2 dental) OR (dental NEAR/2 service*) OR (dental NEAR/1 "check up") OR (dental NEAR/1 (health OR intervention* OR clinic* OR treatment* OR therap* OR program* OR practice* OR education OR procedure* OR restorat* OR regenerative*)) OR (dental stress analys?s) OR denture* OR (oral NEAR/1 care) OR ("dental health" NEAR/1 survey*) OR ((decayed OR missing OR filled) NEAR/2 teeth) OR endodontics OR orthodontics OR periodontics OR prosthodontics OR ((periodont* OR orthodontic* OR prosthodont* OR endodontic) NEAR/1 (treatment* OR procedure* OR surger* OR care)) OR ((dental OR t??th OR periodontal) NEAR/0 cleaning)) OR noft((((orthodontia OR orthodontolog* OR

orthodonty) OR ((oral OR mouth) NEAR/1 surg*) OR ("community periodontal index of treatment needs" OR cpitn) OR ((mouth OR oral) NEAR/1 rehabilitation*) OR (((diagnos* OR examination*) NEAR/0 (oral OR dental))) OR (community NEAR/0 dentistry) OR (((dental OR periodontal OR t??th) NEAR/0 prophylaxis)) OR (((maxillo-mandibular OR maxillomandibular OR oral OR maxillofacial OR orthognathic OR jaw) NEAR/0 surg*) OR ((maxillo-mandibular OR maxillomandibular OR oral OR maxillofacial OR orthognathic) NEAR/1 procedure*)) OR ((oral NEAR/0 hygiene))))) OR noft(((("evidence-based" OR preventative OR cosmetic OR operative OR prosthetic OR reparative) NEAR/1 dentistry) OR (dental NEAR/0 (esthetic* OR aesthetic* OR floss OR scaling* OR brace* OR hygiene* OR polishing OR radiology OR bonding OR soldering* OR prosthetic*))) OR ((dental OR tooth) NEAR/2 filling*) OR ((dental OR tooth OR caries) NEAR/1 prevention) OR (dental NEAR/1 (implant* OR prosthes?s)) OR ((tooth OR teeth) NEAR/0 (whitening OR remineralization* OR polishing OR restoration* OR surger* OR preparation* OR removal*)) OR (dental NEAR/0 infection NEAR/0 control*) OR (tooth NEAR/1 (movement* OR care))) OR noft(fluoridation* OR (oral NEAR/0 medicine) OR (mouth NEAR/0 guard) OR (root NEAR/0 planing) OR ((oral OR maxillofacial) NEAR/2 pathology) OR (periodontal NEAR/0 medicine) OR (periodontal NEAR/0 (prosthesis OR prostheses)) OR (periodontal NEAR/0 prevention) OR (mouth NEAR/0 protection)))

Key

Su = subject heading

Noft = anywhere except full text

NEAR/x = terms within x words of each other (any order)

*= truncation

“ “ = phrase search

? = Mandatory wildcards (stands for exactly 1 character)
