# Supplementary material for: The Impact of Dental Care Programs on Individuals and Their Families: A Scoping Review
Source: Dent J (Basel). 2023 Jan 30;11(2):33. doi: 10.3390/dj11020033 (PMC9954911; doi:10.3390/dj11020033)
Supplement: Supplementary file 1 [file dentistry-11-00033-s001.zip › Supplementary file 2 Outcomes assessed.pdf]

Supplementary file 2. The types of programs and the outcomes assessed categorized by the type of and level of outcomes

Author(s), year	Individual level outcomes							Family level outcomes			
	Dental caries	Gingival health	Oral hygiene	OHRQoL	Oral health knowledge and attitude	Oral health behaviors	Miscellaneous	Parental oral health knowledge and attitude	Parental oral health behaviors	Parent distress and family function	Parental OHRQoL
**Oral Health Education (OHE) programs**											
1. Bizarra et al. 2019[42]		+	0								
2. Faulks et al. 2000[49]					+	+					
3. Freeman et al. 2016[43]				+	+	0					
4. Ganss et al. 2019[44]		+/-	+/-								
5. Henry et al. 2017 (Systematic review)[61]	+										
6. Hoeft et al. 2016*[55]								+	+		
7. Lambert 2019[45]	+		+		+						
8. Livny et al. 2007[52]	0					0					
9. Livny et al. 2008[53]						+					
10. Lopez Cazaux et al. 2019[51]						+					
11. Mariño et al. 2013[46]		+				+					
12. Mariño et al. 2016[47]					+	+					
13. Nakre et al. 2013 (Systematic review) *[60]	+/0	+	+/0		+	+		+	+		
14. Nihtilä et al. 2017[48]			+								
15. Phlypo et al. 2018[37]		0	0		0						
16. Plutzer et al. 2008[36]	+										
17. Sakayori et al. 2016[35]							+ (SF)<br>+ (OF)				

Author(s), year	Individual level outcomes							Family level outcomes			
	Dental caries	Gingival health	Oral hygiene	OHRQoL	Oral health knowledge and attitude	Oral health behaviors	Miscellaneous	Parental oral health knowledge and attitude	Parental oral health behaviors	Parent distress and family function	Parental OHRQoL
18. Samson et al. 2009[34]			+/0								
19. Schulz-Weidner et al. 2021[56]		**+**	**+**								
20. Seo and Kim 2020 (Systematic review)[57]				+			+ (SF)				
21. Sfeatcu et al. 2019[33]	+	+			+	+					
22. Smith et al. 2018[54]	+										
23. Stein et al. 2016 (Systematic review)[58]	+/0	+/0	+/0		+	+					
24. Tubert-Jeannin et al. 2012[41]	+/-										
25. Ueno et al. 2012[40]		+	+				+ (M)				
26. Van den Branden et al. 2014[38]					+/0						
27. Wagner et al. 2016[39]	+										
28. Wang et al. 2015 (Systematic review)[59]			+								
29. Winter et al. 2018[50]					+	+					
**Diagnostic and preventive programs**											
1. Achembong et al. 2014[66]	+										
2. Andruškevičienė et al. 2008[78]	+		+								

Author(s), year	Individual level outcomes							Family level outcomes			
	Dental caries	Gingival health	Oral hygiene	OHRQoL	Oral health knowledge and attitude	Oral health behaviors	Miscellaneous	Parental oral health knowledge and attitude	Parental oral health behaviors	Parent distress and family function	Parental OHRQoL
3. Bergström et al. 2015[77]	+										
4. Burgette et al. 2017*[92]				+						+	
5. Dohnke - Hohrmann et al. 2004[67]	+										
6. Dudovitz et al. 2018[89]	+				0						
7. Eckersten et al. 2010[97]							0 (F)				
8. García-Pola et al. 2021[94]	+										
9. Källestål 2005[69]	+										
10. Kim et al. 2017[93]			+								
11. Lai et al. 2018[90]	+										
12. Lee et al. 2017[83]		+									
13. Lee et al. 2021[95]	0	**+**	**+**				+ (SF)<br>+ (QoL)				
14. Lin et al. 2011[91]		+			+	+					
15. Macnab et al. 2008[76]	+					+					
16. Meyer et al. 2014[79]	+										
17. Milsom et al. 2014[68]	+										
18. Sköld et al. 2005[84]	+										
19. Muller-Bolla et al. 2016[88]	+										
20. Nakamura et al. 2009[82]	+										
21. Pieper et al. 2013[71]	+										
22. Ruff et al. 2018[85]	+										
23. Schroth et al. 2015*[87]	+				+	+		+	+/0		

Author(s), year	Individual level outcomes							Family level outcomes			
	Dental caries	Gingival health	Oral hygiene	OHRQoL	Oral health knowledge and attitude	Oral health behaviors	Miscellaneous	Parental oral health knowledge and attitude	Parental oral health behaviors	Parent distress and family function	Parental OHRQoL
24. Simmer-Beck et al. 2015[86]	+										
25. Sköld et al. 2016[64]	+										
26. Slade et al. 2011[63]	+										
27. Sloane et al. 2013[80]		+	+								
28. Starr et al. 2021[96]	+										
29. Sundell et al. 2013[70]	0										
30. Wagner et al. 2017[65]	+										
31. Wennhall et al. 2005[81]	+					+					
32. Wennhall et al. 2008[73]	+	0	0			0					
33. Winter et al. 2016[75]	+										
34. Winter et al. 2018[74]	+										
35. Zimmer et al. 2001[72]	0/+										
**Interventional programs**											
1. Alsumiat et al. 2015[98]				0	0	0					
2. Alsumiat et al. 2019*[99]	+			0				0	0		0
3. George et al. 2018[106]	+	+	+		+	+					
4. Gomez et al. 2001[105]	+										
5. Hyde et al. 2005[103]						+					
6. Hyde et al. 2006[104]				+							
7. Janssens et al. 2018[102]	+						+ (OHS)				
8. Larsen et al. 2016[107]	+										

Author(s), year	Individual level outcomes							Family level outcomes			
	Dental caries	Gingival health	Oral hygiene	OHRQoL	Oral health knowledge and attitude	Oral health behaviors	Miscellaneous	Parental oral health knowledge and attitude	Parental oral health behaviors	Parent distress and family function	Parental OHRQoL
9. Ortuno Borroto et al. 2021[109]				**+**							
10. Rong et al. 2009[108]	+	+		0							
11. Walker et al. 2007[101]				+							
12. Wyatt et al. 2009[100]							+ (COD)				

Miscellaneous outcomes include Salivary Flow/Salivary Secretion (SF), Malodor (M), Fluorosis (F), Oral Function (OF), Quality of Life (QoL), Oral Health Stability (OHS), and Clinical Oral Disorder (COD).

+ indicates a favorable outcome
- indicates an unfavorable outcome
0 indicates no change
* indicates that the study addresses a family level outcome
