# Supplementary material for: The Impact of Dental Care Programs on Individuals and Their Families: A Scoping Review
Source: Dent J (Basel). 2023 Jan 30;11(2):33. doi: 10.3390/dj11020033 (PMC9954911; doi:10.3390/dj11020033)
Supplement: Supplementary file 1 [file dentistry-11-00033-s001.zip › Supplementary file 3 Program setting classification.pdf]

Supplementary file 3. Program setting classification

Author(s), year	Country	Study design	Personnel category	Target group category	Program type	Follow-up period
**School based setting**						
1. Alsumiat et al. 2015[98]	Kuwait	Cross-sectional study	N/A	Adolescents	Interventional	N/A
2. Alsumiat et al. 2019[99]	Kuwait	Cross-sectional study	Dental personnel	Children	Interventional	3 years
3. Andruškevičienė et al. 2008[78]	Lithuania	Experimental study	Non-healthcare personnel	Children	Diagnostic and preventive	3 years
4. Bergström et al. 2015[77]	Sweden	Retrospective study	• Healthcare personnel<br>• Dental personnel	Children	Diagnostic and preventive	4 years
5. Burgette et al. 2017[92]	United States	Experimental study	N/A	Children	Diagnostic and preventive	2 years
6. Dohnke – Hohrmann et al. 2004[67]	Germany	Experimental study	N/A	Children	Diagnostic and preventive	4 years
7. Dudovitz et al. 2018[89]	United States	Cross-sectional study	Dental personnel	Children	Diagnostic and preventive	1 year
8. Freeman et al. 2016[43]	Ireland	Experimental study	• Non-healthcare personnel<br>• Dental personnel	Children	OHE	1 year
9. Lambert et al. 2019[45]	Belgium	Experimental study	Dental personnel	Children	OHE	4 years
10. Livny et al. 2008[53]	Israel	Experimental study	Dental personnel	Children	OHE	2 years
11. Lopez Cazaux et al. 2019[51]	France	Experimental study	Dental personnel	Children and Adolescents	OHE	8 months
12. Macnab et al. 2008[76]	Canada	Cross-sectional study	• Dental personnel	Adolescents	Diagnostic and preventive	3 years

Author(s), year	Country	Study design	Personnel category	Target group category	Program type	Follow-up period
			• Healthcare personnel			
13. Sköld et al. 2005[84]	Sweden	Experimental study	Dental personnel	Adolescents	Diagnostic and preventive	3 years
14. Muller-Bolla et al. 2016[88]	France	Experimental study	Dental personnel	Children	Diagnostic and preventive	3 years
15. Nakamura et al. 2009[82]	Japan	Experimental study	N/A	Children	Diagnostic and preventive	5 years
16. Pieper et al. 2013[71]	Germany	Not specified	N/A	Adolescents	Diagnostic and preventive	N/A
17. Ruff et al. 2018[85]	United States	Longitudinal study	Dental personnel	Children	Diagnostic and preventive	10 years
18. Schulz-Weidner et al. 2021ɫ[56]	Germany	Experimental study	• Non-health care personnel<br>• Dental personnel	Children	OHE	3 and 6 months
19. Sfeatcu et al. 2018[33]	Romania	Experimental study	Dental personnel	Adolescents	OHE	2 years
20. Simmer-Beck et al. 2015[86]	United States	Secondary analyses of administrative data	Dental personnel	Children	Diagnostic and preventive	N/A
21. Sköld 2016[64]	Sweden	Experimental study	Dental personnel	Adolescents	Diagnostic and preventive	3 years
22. Starr et al. 2021[92]	United States	Prospective cohort	Dental personnel	Children	Diagnostic and preventive	6 years
23. Sundell et al. 2013[70]	Sweden	Experimental study	Dental personnel	Children	Diagnostic and preventive	2 years
24. Tubert-Jeannin et al. 2012[41]	France	Cross-sectional study	Dental personnel	Children	OHE	1-4 years
25. Ueno et al. 2012[40]	Japan	Experimental study	Dental personnel	Adolescents	OHE	1 year
26. Winter et al. 2016[75]	Germany	Longitudinal study	N/A	Children	Diagnostic and preventive	4 years

Author(s), year	Country	Study design	Personnel category	Target group category	Program type	Follow-up period
27. Winter et al. 2018[74]	Germany	Experimental study	N/A	Children	Diagnostic and preventive	2 years
28. Zimmer et al. 2001[72]	Germany	Experimental study	Dental personnel	Children	Diagnostic and preventive	2 years
**Long term care and institutional settings**						
1. Bizarra et al. 2019[42]	Portugal	Experimental study	Non-healthcare personnel	N/A	OHE	6 months
2. Janssens et al. 2018[102]	Belgium	Longitudinal study	• Healthcare personnel<br>• Dental personnel	Elders	Interventional	22.5 months
3. Phlypo et al. 2018[37]	Belgium	Experimental study	Non-healthcare personnel	N/A	OHE	5 weeks
4. Rong et al. 2009[108]	Hong Kong	Experimental study	Dental personnel	Elders	Interventional	6 months
5. Samson et al. 2009[34]	Norway	Experimental study	Healthcare personnel	N/A	OHE	6 years
6. Sloane et al. 2013[80]	United States	Experimental study	Healthcare personnel	N/A	Diagnostic and preventive	8 weeks
7. Walker et al. 2007[101]	United States	Experimental study	Dental personnel	Elders	Interventional	2 weeks-6 months
8. Wyatt et al. 2009[100]	Canada	Experimental study	Dental personnel	Elders	Interventional	5 years
**Medical setting**						
1. Achembong et al. 2014[66]	United States	Ecological study	Healthcare personnel	Children	Diagnostic and preventive	N/A
2. George et al. 2018[106]	Australia	Experimental study	• Healthcare personnel<br>• Dental personnel	Adults	Interventional	N/A
3. Gomez et al. 2001[105]	Chile	Experimental study	Dental personnel	Children	Interventional	4 years

Author(s), year	Country	Study design	Personnel category	Target group category	Program type	Follow-up period
4. Kim et al. 2017[93]	South Korea	Experimental study	• Non-healthcare personnel<br>• Dental personnel	Adults and Elders	Diagnostic and preventive	8 weeks
5. Lai et al. 2018*[90]	Singapore	Experimental study	Dental personnel	Children	Diagnostic and preventive	2 years
6. Lee et al. 2021[95]	Korea	Experimental study	Dental personnel	Adults and Elders	Diagnostic and preventive	6 months
7. Meyer et al. 2014[79]	Germany	Experimental study	Dental personnel	Children and adolescents	Diagnostic and preventive	18-19 years
8. Plutzer et al. 2008[36]	Australia	Experimental study	N/A	Children	OHE	1.5 years
9. Schulz-Weidner et al. 2021ƭ[56]	Germany	Experimental study	• Non-health care personnel<br>• Dental personnel	Children	OHE	3 and 6 months
10. Wagner et al. 2016[39]	Austria	Prospective cohort	Dental personnel	Children	OHE	5 years
11. Winter et al. 2018[50]	Germany	Experimental study	• Non-healthcare personnel<br>• Healthcare personnel<br>• Dental personnel	Children	OHE	N/A
**Community-based setting/Public health setting**						
1. Eckersten et al. 2010[97]	Sweden	Retrospective study	Dental personnel	Children	Diagnostic and preventive	5-6 years
2. Faulks et al. 2000[49]	France	Experimental study	Dental personnel	Adults	OHE	9-12 months
3. Hoeft et al. 2016[55]	United States	Experimental study	Non-healthcare personnel	Adults	OHE	3 months

Author(s), year	Country	Study design	Personnel category	Target group category	Program type	Follow-up period
4. Hyde et al. 2005[103]	United States	Experimental study	Dental personnel	Adults	Interventional	18 months
5. Hyde et al. 2006[104]	United States	Experimental study	Dental personnel	Adults	Interventional	18 months
6. Källestål 2005[69]	Sweden	Experimental study	Dental personnel	Adolescents	Diagnostic and preventive	5 years
7. Lee et al. 2017[83]	South Korea	Experimental study	Dental personnel	Adults and Elders	Diagnostic and preventive	2 months
8. Livny et al. 2007[52]	Israel	Experimental study	Healthcare personnel	Children	OHE	2 years
9. Mariño et al. 2013[46]	Australia	Experimental study	N/A	Elders	OHE	16 weeks
10. Mariño et al. 2016[47]	Australia	Experimental study	N/A	Adults and elders	OHE	N/A
11. Nihtilä et al. 2017[48]	Finland	Experimental study	• Non-healthcare personnel<br>• Healthcare personnel	Elders	OHE	6 months
12. Ortuno Borroto et al. 2021Ƨ[109]	Chile	Prospective cohort	Dental personnel	Adults and Elders	Interventional	N/A
13. Sakayori et al. 2016[35]	Japan	Not specified	Dental personnel	Elders	OHE	1 year
14. Schroth et al. 2015[87]	Canada	Cross-sectional study	Dental personnel	Children	Diagnostic and preventive	N/A
15. Slade et al. 2011[63]	Australia	Experimental study	• Healthcare personnel<br>• Dental personnel	Children	Diagnostic and preventive	2 years
16. Smith et al. 2018[54]	Australia	Experimental study	• Healthcare personnel	Children	OHE	30 months

Author(s), year	Country	Study design	Personnel category	Target group category	Program type	Follow-up period
			• Dental personnel			
17. Van den Branden et al. 2014[38]	Belgium	Experimental study	Healthcare personnel	Children	OHE	3 and 5 years
18. Wagner et al. 2017[65]	Germany	Longitudinal study	• Non-healthcare personnel<br>• Healthcare personnel	Children	Diagnostic and preventive	5 years
19. Wennhall et al. 2005[81]	Sweden	Experimental study	Dental personnel	Children	Diagnostic and preventive	1 year
20. Wennhall et al. 2008[73]	Sweden	Experimental study	Dental personnel	Children	Diagnostic and preventive	5 years
**Dental settings**						
1. Ganss et al. 2019[44]	Germany	Retrospective study	Dental personnel	Adults	OHE	15 years
2. García-Pola et al. 2021[94]	Spain	Prospective cohort	Dental personnel	Children	Diagnostic and preventive	6 years
3. Lai et al. 2018*[90]	Singapore	Experimental study	Dental personnel	Children	Diagnostic and preventive	2 years
4. Larsen et al. 2016[107]	United States	Retrospective data analyses of administrative data	Dental personnel	Children	Interventional	2 years
5. Lin et al. 2011[91]	Canada	Longitudinal study	Dental personnel	Adults	Diagnostic and preventive	1 year
6. Milsom et al. 2014[68]	UK	Retrospective study using administrative data	Dental personnel	Children	Diagnostic and preventive	4 years
7. Ortuno Borroto et al. 2021Ƨ[109]	Chile	Prospective cohort	Dental personnel	Adults and Elders	Interventional	N/A
**Reviews**						
1. Henry et al. 2017[61]	Not applicable	Systematic review	N/A	Children and adults	OHE	N/A

Author(s), year	Country	Study design	Personnel category	Target group category	Program type	Follow-up period
2. Nakre et al. 2013[60]	Not applicable	Systematic review	Non-healthcare personnel	Children, adults, elders	OHE	N/A
3. Seo and Kim 2020[57]	Not applicable	Systematic review	• Healthcare personnel<br>• Dental personnel	Elders	OHE	8 weeks – 3 months
4. Stein et al. 2016[58]	Not applicable	Systematic review	Dental personnel	Children and Adolescents	OHE	N/A
5. Wang et al. 2015[59]	Not applicable	Systematic review	Dental personnel	Elders	OHE	N/A

* Reported in medical and dental settings, ϯ Reported in school and medical settings, Ꞓ Reported in Community-based setting and dental settings
